# GS-DeepNet: mastering tokamak plasma equilibria with deep neural networks and the Grad–Shafranov equation

**DOI:** 10.1038/s41598-023-42991-5

**Published:** 2023-09-22

**Authors:** Semin Joung, Y.-C. Ghim, Jaewook Kim, Sehyun Kwak, Daeho Kwon, C. Sung, D. Kim, Hyun-Seok Kim, J. G. Bak, S. W. Yoon

**Affiliations:** 1 Department of Nuclear and Quantum Engineering, KAIST, Daejeon, 34141 South Korea; 2 Korea Institute of Fusion Energy, Daejeon, 34133 South Korea; 3 Max-Planck-Institute Fur Plasmaphysik, 17491 Greifswald, Germany; 4 Mobiis Co., Ltd., Seongnam-Si, Gyeonggi-Do 13486 South Korea; 5 University of Wisconsin–Madison, Madison, WI 53706 USA

**Keywords:** Nuclear fusion and fission, Magnetically confined plasmas

## Abstract

The force-balanced state of magnetically confined plasmas heated up to 100 million degrees Celsius must be sustained long enough to achieve a burning-plasma state, such as in the case of ITER, a fusion reactor that promises a net energy gain. This force balance between the Lorentz force and the pressure gradient force, known as a plasma equilibrium, can be theoretically portrayed together with Maxwell’s equations as plasmas are collections of charged particles. Nevertheless, identifying the plasma equilibrium in real time is challenging owing to its free-boundary and ill-posed conditions, which conventionally involves iterative numerical approach with a certain degree of subjective human decisions such as including or excluding certain magnetic measurements to achieve numerical convergence on the solution as well as to avoid unphysical solutions. Here, we introduce GS-DeepNet, which learns plasma equilibria through solely unsupervised learning, without using traditional numerical algorithms. GS-DeepNet includes two neural networks and teaches itself. One neural network generates a possible candidate of an equilibrium following Maxwell’s equations and is taught by the other network satisfying the force balance under the equilibrium. Measurements constrain both networks. Our GS-DeepNet achieves reliable equilibria with uncertainties in contrast with existing methods, leading to possible better control of fusion-grade plasmas.

## Introduction

The ultimate goal of scientific and engineering research in the field of nuclear fusion is to build a power plant producing sustainable and clean electricity through fusion reactions from a confined plasma heated up to ~ 100 million degrees Celsius^1,2^. A tokamak is a torus-shape vacuum vessel within which the plasma is confined by magnetic fields directed along the long (toroidal) and short (poloidal) ways around the torus. Maintaining such a high-temperature plasma for a long period of time (e.g., longer than 400 s^3^) requires the plasma pressure gradient and the Lorentz force to be balanced throughout the plasma volume^4^ during operation of a tokamak. This means that knowing internal spatial structures of the plasma pressure and the magnetic fields in real-time is beneficial for operating tokamak plasmas.

Direct in situ measurements of the plasma structures are often difficult to make owing to the harsh environment; e.g., environments with high temperature and radiation. Although optics systems that directly measure internal information such as the electron temperature and density^5^ and magnetic pitch angle^6^ exist, these measurements are spatially localized and require a magnetic field structure to be mapped onto the whole plasma volume. Hence, a suite of magnetic diagnostics^7^, fundamental measurement devices installed behind the plasma-facing components far from the plasma, is used to obtain the magnetic field structures indirectly by solving the Grad–Shafranov (GS) equation^8,9^. The GS equation constrained with the measured magnetic fields describes a force balanced plasma state conforming to Maxwell’s equations with a toroidal axisymmetry assumption, and finding a solution to the GS equation under such constraints is thus regarded as reconstructing the magnetohydrodynamic (MHD) equilibrium of a toroidal plasma.

The GS equation, resembling the Hicks equation^10^ that describes an axisymmetric inviscid fluid, is a two-dimensional (poloidal cross-section), nonlinear, elliptic partial differential equation. Owing to the nonlinearity of the GS equation, reconstructing the MHD equilibrium consistent with the GS equation traditionally requires an iterative numerical approach as it is both an inverse and a free-boundary problem. Only external measurements of the magnetic fields are typically available, and we do not have a priori knowledge of where the boundary separates the vacuum region from the plasma region. These difficulties hinder the real-time application of the GS equation. Of course, a simple solution for real-time application is to sacrifice the accuracy of the solution as in Ref.^11^. However, even if accuracy is eschewed, human expert choices are made in reaching numerical convergence. Traditional numerical algorithms of the reconstruction, chiefly EFIT^12^, often require subjective decisions in the manual selection of magnetic measurements. The neglected data do not participate in reconstructing a plasma equilibrium because they tend to obstruct the search for a converging numerical solution.

Attempts have been made to parallelize the traditional numerical algorithms using graphical processing units (GPUs)^13,14^ or a supervised deep neural network^15^, which fulfill the real-time demand but still require human intervention because they are based on the EFIT algorithm. In contrast, reconstruction methods using Bayesian inference^16–18^ have been introduced to eliminate (or at least explicitly articulate) manual selections, but they are unlikely to be used for real-time purposes owing to their heavy computations. We note that reconstructing more detailed plasma equilibria in real time using internal information is an active research area^19,20^.

Recent scientific computing has been supported by deep learning^21^, and therefore various approaches have been proposed for neural networks to learn physics-based differential equations, such as in solving the many-electron Schrödinger equation^22,23^, the Navier–Stokes equation^24^ and an atmospheric model for climate modeling^25^. Other examples include interpolating partial differential equations^26–28^ and regularizing neural networks with the Kohn − Sham equations^29^. These previous works require actual solutions^27,28^, prior knowledge on some of the unknown parameters^24–26^, or approximated solution states^22,23,29^ of the target governing equations. Similarly, attempts have been made to find a solution to the GS equation using neural networks^26,30^, and they work with given internal profiles since they are GS equation solvers, i.e., not an equilibrium reconstruction algorithm. Additionally, there is a method^31^ of using neural networks to solve a Stefan problem^32^, which is a free-boundary problem and describes a phase-change between liquid and solid states. However, the method assumes that the boundary of the phase-change between the states is already known.

We propose an algorithm, Grad–Shafranov Deep Neural Networks (GS-DeepNet), which learns plasma equilibria without using existing traditional numerical algorithms that reconstruct the equilibria (i.e., find the solution to the GS equation). First and foremost, GS-DeepNet is trained through self-teaching unsupervised learning, without any guess of the solutions. The only known information is the GS equation and the externally measured magnetic fields, acting as the boundary condition of the differential equation, with no manual selections. Second, GS-DeepNet uses typical fully-connected neural networks known to be capable of retaining real-time application. Finally, GS-DeepNet uses an auxiliary module that detects plasma boundary information solely from network outputs. To reach these outcomes, we develop neural networks that are capable of solving a nonlinear elliptic partial differential equation under free-boundary and inverse conditions, namely the GS equation. GS-DeepNet is trained, validated and tested in the Korea Superconducting Tokamak Advanced Research (KSTAR) environment^33^.

## Results

### Architecture of GS-DeepNet

**Figure 1:**
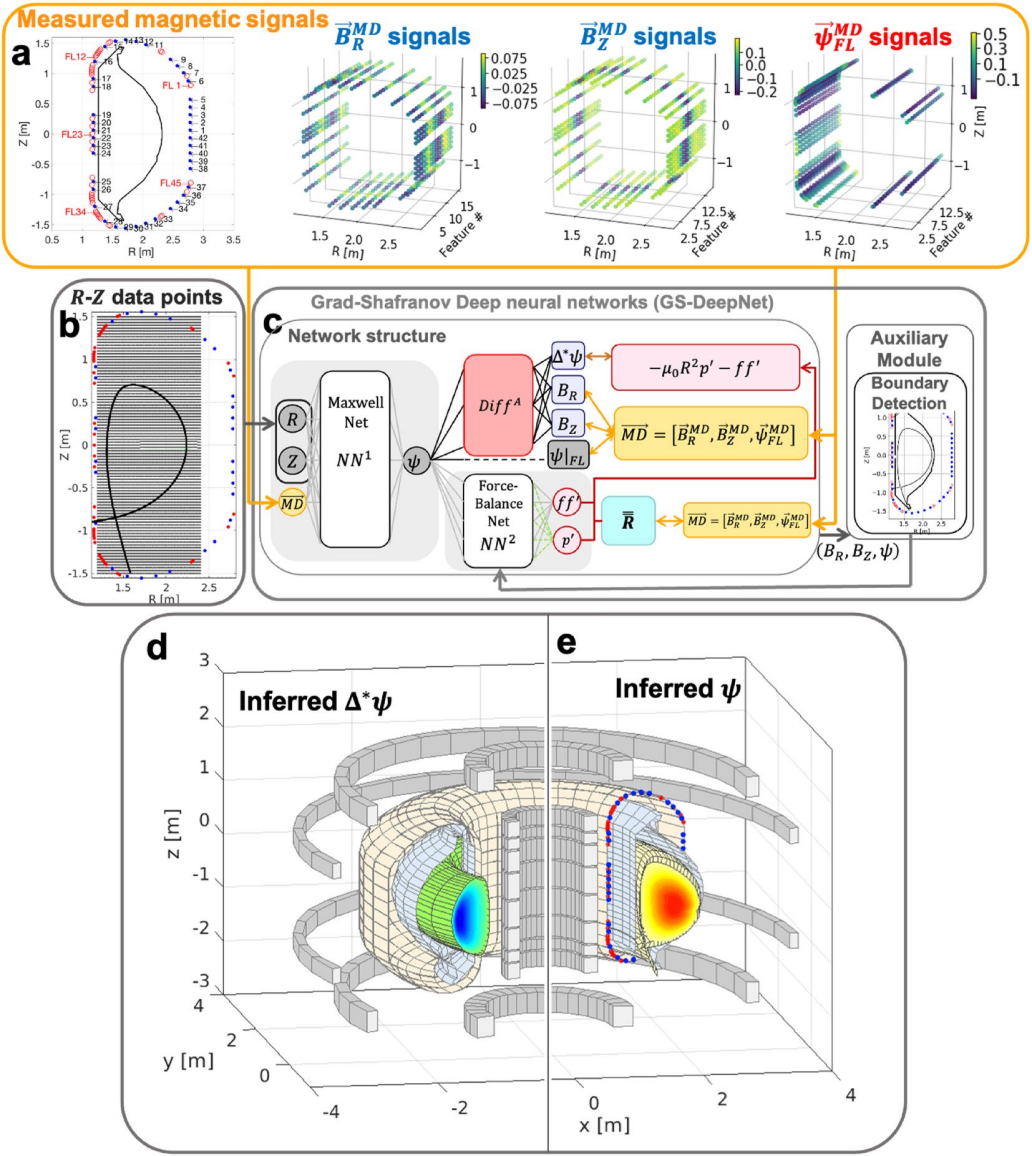
Self-teaching unsupervised learning scheme of GS-DeepNet. (**a**) Leftmost: locations and sensor numbers of the KSTAR magnetic diagnostics. There are 42 magnetic pick-up probes each measuring ${B}_{R}$ and ${B}_{Z}$ (blue) and 45 flux loops measuring ${\psi }_{FL}$ (red). The plots of the ${\vec{B}}_{R}^{MD}$, ${\vec{B}}_{Z}^{MD}$ and ${\vec{\psi }}_{FL}^{MD}$ signals show examples of measurements made using 31 pick-up probes and 45 flux loops on the poloidal cross-section $(R, Z)$ space as a function of time, which we refer to as the feature. Colors represent magnitude of the measurements. (**b**) Black dots: $41\times 41$ grid points where GS-DeepNet finds a solution. Blue and red dots: positions of the pick-up probes and flux loops as in (**a**). Black line: example of the plasma boundary dividing the plasma region (inside the line) from a vacuum area. This boundary is known as the last closed flux surface. (**c**) Schematic representation of GS-DeepNet. The network $N{N}^{1}$ (Maxwell Net) takes a spatial position and a feature of the magnetic data as its input and outputs a poloidal flux function that is used to calculate ${B}_{R}$, ${B}_{Z}$ and ${\Delta }^{*}\psi$ with the automatic differential operator $Dif{f}^{A}$. After determining a plasma boundary using the auxiliary module with the output of the Maxwell Net, the network $N{N}^{2}$ (Force-Balance Net) takes a poloidal flux function as its input and outputs a pair $\left({p}{^\prime},{ff}{^\prime}\right)$. (**d**,**e**) Example of the three-dimensional configurations of the reconstructed ${\Delta }^{*}\psi$ (d) and $\psi$ (e) from GS-DeepNet. KSTAR poloidal field coils (gray), the vacuum vessel wall (light orange) and the plasma facing components (light blue) are also shown as well as the magnetic diagnostics (blue and red dots).

Our novel unsupervised learning algorithm GS-DeepNet, comprising two deep neural networks $N{N}_{\Theta }^{1}$ and $N{N}_{\theta }^{2}$ (Fig. 1c) with parameters $\Theta$ and $\theta$, respectively, finds a solution of the GS equation, which is

1 $${\Delta }^{*}\psi =-{\mu }_{0}R{J}_{\phi }=-{\mu }_{0}{R}^{2}\frac{dp\left(\psi \right)}{d\psi }-f\left(\psi \right)\frac{df\left(\psi \right)}{d\psi },$$

where ${\Delta }^{*}\equiv R\frac{\partial }{\partial R}\frac{1}{R}\frac{\partial }{\partial R}+\frac{{\partial }^{2}}{\partial {Z}^{2}}$ is defined as a two-dimensional, elliptic partial differential operator in the tokamak machine coordinates $\left(R, \phi , Z\right)$ with the toroidal axisymmetry assumption^8,9^; i.e., $\frac{\partial }{\partial \phi }=0$. The first line of Eq. (1) is a consequence of $\nabla \cdot \vec{B}=0$ and $\nabla \times \vec{B}={\mu }_{0}\vec{J}$ in an axisymmetric toroidal geometry, where $\vec{B}$ and $\vec{J}$ are respectively the magnetic field and current density from Maxwell’s equations. It states that the elliptic differential operator acting on the poloidal flux function $\psi$ is proportional to the product of the major radius $R$ and the toroidal plasma current density ${J}_{\phi }$ with the permeability ${\mu }_{0}$ as the constant of proportionality. The second line of Eq. (1) can be derived using the force-balanced MHD momentum equation, $\vec{J}\times \vec{B}=\nabla p$, where $p$ is the plasma pressure. The poloidal current function $f=R {B}_{\phi }$ with toroidal magnetic field ${B}_{\phi }$ is a quantity related to the poloidal plasma current. Formally, $p$ and $f$ are functions of only $\psi$. Thus, the task of GS-DeepNet is to find $\psi (R, Z)$ in the two-dimensional poloidal plane (Fig. 1b) with two free functions $p(\psi )$ and $f(\psi )$ while satisfying the boundary conditions set by a given magnetic measurement state that we call a feature. Note that finding $\psi (R, Z)$ together with $p(\psi )$ and $f(\psi )$ is what is known as the reconstruction of the MHD equilibrium of a toroidal plasma.

With a single spatial point $\left(R, Z\right)$ and a set of magnetic measurements  $\overrightarrow{MD}=\left({\vec{B}}_{R}^{MD}, {\vec{B}}_{Z}^{MD},{\vec{\psi }}_{FL}^{MD}\right)$ as inputs, $N{N}_{\Theta }^{1}$ is trained to output a single-valued flux function, $\psi =N{N}_{\Theta }^{1}\left(R, Z,\overrightarrow{MD}\right)$. The vector notation (${\vec{B}}_{R(Z)}^{MD}$ or ${\vec{\psi }}_{FL}^{MD}$) means a collection of measurements made at different spatial locations in a single time slice (i.e., a feature), namely 31 measurements of ${B}_{R}^{MD}$, 31 of ${B}_{Z}^{MD}$ and 45 of ${\psi }_{FL}^{MD}$, resulting in a total of 107 magnetic measurements. Here, ${B}_{R}^{MD}$ and ${B}_{Z}^{MD}$ are respectively the $R$- and $Z$-components of the poloidal magnetic field, measured using a magnetic pick-up probe (blue dots in Fig. 1a,b). ${\psi }_{FL}^{MD}$ is the poloidal magnetic flux measured using a magnetic flux loop (red open circles in Fig. 1a or red dots in Fig. 1b). These magnetic pick-up probes and flux loops installed at the tokamak boundary constitute a suite of magnetic diagnostics and impose the boundary conditions in real time on GS-DeepNet. The magnetic pick-up probes measure the normal (${B}_{n}^{MD}$) and tangential (${B}_{t}^{MD}$) components of the poloidal magnetic fields with respect to the vacuum vessel wall where the probes are installed, and ${B}_{R}^{MD}$ and ${B}_{Z}^{MD}$ are thus calculated using a simple coordinate conversion (see Fig. S1). We specified $41\times 41$ grid points (black dots in Fig. 1b) on the $\left(R, Z\right)$ plane (i.e., on a poloidal plane), where $N{N}_{\Theta }^{1}$ is trained to output the values of $\psi$. The GS equation constrains only the derivative of $\psi$, and we must therefore supply proper boundary conditions as discussed in Supplementary Note S1. For this reason, we let $N{N}_{\Theta }^{1}$ also accept the $\left(R, Z\right)$ positions of the magnetic diagnostics as an input and be trained to provide values of $\psi$ at the tokamak boundary. Thus, the solution space is the $41\times 41$ grid points and the positions of the magnetic diagnostics. Using the automatic differential operator^34^ ($Dif{f}^{A}$ in Fig. 1c) and the flux function $\psi$, ${B}_{R}(=-1/R\cdot \partial \psi /\partial Z)$, ${B}_{Z}(=1/R\cdot \partial \psi /\partial R)$ and ${\Delta }^{*}\psi$ are calculated and used to train the network with the measured boundary conditions. We refer to $N{N}_{\Theta }^{1}$ together with $Dif{f}^{A}$ as the Maxwell Net.

The output of $N{N}_{\Theta }^{1}$, $\psi$, is fed into $N{N}_{\theta }^{2}$ , which outputs two free functions: $\left(dp/d\psi ,f df/d\psi \right)\equiv \left({p}{^\prime},{ff}{^\prime}\right)=N{N}_{\theta }^{2}\left(\psi \right)$. The input of $N{N}_{\theta }^{2}$ is $\psi$, and the two free functions are thus guaranteed to be functions of only $\psi$. Tokamak plasmas have a well-defined plasma boundary known as the last closed flux surface (LCFS, thick black line in Fig. 1b); consequently, outside the LCFS, the toroidal plasma current density ${J}_{\phi }$ is set to be zero based on a common assumption, resulting in ${\Delta }^{*}{\psi }_{out}=0$ from Eq. (1), where ${\psi }_{out(in)}$ is the flux function $\psi$ outside (inside) the LCFS. Thus, finding $\left({p}{^\prime},{ff}{^\prime}\right)$ consistent with Eq. (1) is relevant only inside the LCFS. Unfortunately, the LCFS cannot be identified until $\psi$’s in the solution space are known, which is the reason why solving the GS equation is a free-boundary problem. We note that there is a recent work to predict the future LCFSs in real-time with deep learning^35^, and it requires a free-boundary problem solver, e.g., EFIT, to identify where the boundary locations are. GS-DeepNet first infers the LCFS on the basis of $\left({B}_{R},{B}_{Z},\psi \right)$ in the solution space from $N{N}_{\Theta }^{1}$ using an auxiliary module for the boundary detection (Fig. 1c) (see “Methods”). $N{N}_{\theta }^{2}$ then outputs $\left({p}{^\prime},{ff}{^\prime}\right)$ using the normalized input $\psi$ if it is inside the LCFS. Note that $\psi$ is normalized to be zero at the magnetic axis and 1 at the LCFS according to the boundary detection module. The output $\left({p}{^\prime},{ff}{^\prime}\right)$ is used to calculate $-{\mu }_{0}{R}^{2}{p}{^\prime}-{ff}{^\prime}$ , which must be equal to ${\Delta }^{*}{\psi }_{in}$ according to the second line of Eq. (1), which is the reason why we refer to $N{N}_{\theta }^{2}$ as the Force-Balance Net. As mentioned earlier, $p(\psi )$ and $f(\psi )$ are free functions, and they thus have large numbers of degrees of freedom. To constrain these free functions (i.e., to train the $N{N}_{\theta }^{2}$ network) such that they convey physical meanings, as they should, we use the fact that ${\mu }_{0}{R}^{2}{p}{^\prime}+{ff}{^\prime}={\mu }_{0}R{J}_{\phi }$ from Eq. (1). More specifically, $-{\mu }_{0}{R}^{2}{p}{^\prime}-{ff}{^\prime}$, which dictates what the current source ${J}_{\phi }$ has to be inside the LCFS, must be constrained with the given magnetic data $\overrightarrow{MD}$, (i.e., a feature) via a response matrix $\overline{\overline{\mathfrak{R}}}$ calculated using the Biot–Savart law (see “Methods”) and the measured total plasma current ${I}_{m}^{plasma}$ , which is a quantity routinely measured using a Rogowski coil in real time^7^.

In summarizing how the unsupervised GS-DeepNet works, the Maxwell Net $N{N}_{\Theta }^{1}$ with the input of $(R,Z, \overrightarrow{MD})$ generates $\psi$, whose normalized value is the input to the Force-Balance Net $N{N}_{\theta }^{2}$ generating $\left({p}{^\prime},{ff}{^\prime}\right)$. Because ${\Delta }^{*}\psi$ (from the Maxwell Net) must be equal to $-{\mu }_{0}{R}^{2}\cdot {p}{^\prime}-{ff}{^\prime}$ (from the Force-Balance Net), GS-DeepNet trains itself until the Maxwell Net and Force-Balance Net are consistent with each other while matching the measured magnetic signals $\overrightarrow{MD}$ at the tokamak boundary using the automatic differential operator for the Maxwell Net and the response matrix $\overline{\overline{\mathfrak{R}}}$ for the Force-Balance Net. Of course, we impose that there is no plasma current outside the LCFS, where the LCFS is determined by the plasma boundary detection module; thus, the Maxwell Net is also trained to give ${\psi }_{out}$ such that ${\Delta }^{*}{\psi }_{out}=0$. This constraint brings an ill-posed condition in our problem, which is explained in detail in Methods. To resolve such an issue, we train the Force-Balance Net using the concept of transfer learning^36^ and the singular value decomposition (SVD) technique (see “Methods”) in addition to a usual gradient descent algorithm which makes our algorithm novel.

Both the Maxwell Net and Force-Balance Net have multiple fully-connected layers^21^ with dropout^37^ and swish nonlinear activation functions^38^. The Maxwell Net has three hidden layers with 100 neurons and a bias for each layer, whereas the Force-Balance Net has two hidden layers with 60 and 6 nodes for the first and second layers, respectively, without any bias nodes. We used a dropout scheme with a rate of 0.05 and 0.10 for the Maxwell Net and Force-Balance Net, respectively, during the training process. The same dropout rates are also used for both networks during the test (prediction) phase to obtain the model uncertainty^39^. A detailed description on how we train GS-DeepNet such as collecting dataset and defining loss functions is provided in “Methods”.

As an example, two-dimensional configurations of ${\Delta }^{*}\psi$ and $\psi$ from GS-DeepNet are shown in Fig. 1d and e, respectively, with some relevant tokamak structures of KSTAR^33^ for this work. Tokamak plasmas are toroidally axisymmetric as assumed in the GS equation, and ${\Delta }^{*}\psi$ and $\psi$ on a two-dimensional poloidal $(R, Z)$ plane thus suffice for us to reconstruct an axisymmetry two-dimensional structure of the MHD equilibrium of a tokamak plasma.

### Statistical analysis of GS-DeepNet training: how well GS-DeepNet teaches itself to solve the GS equation

**Figure 2:**
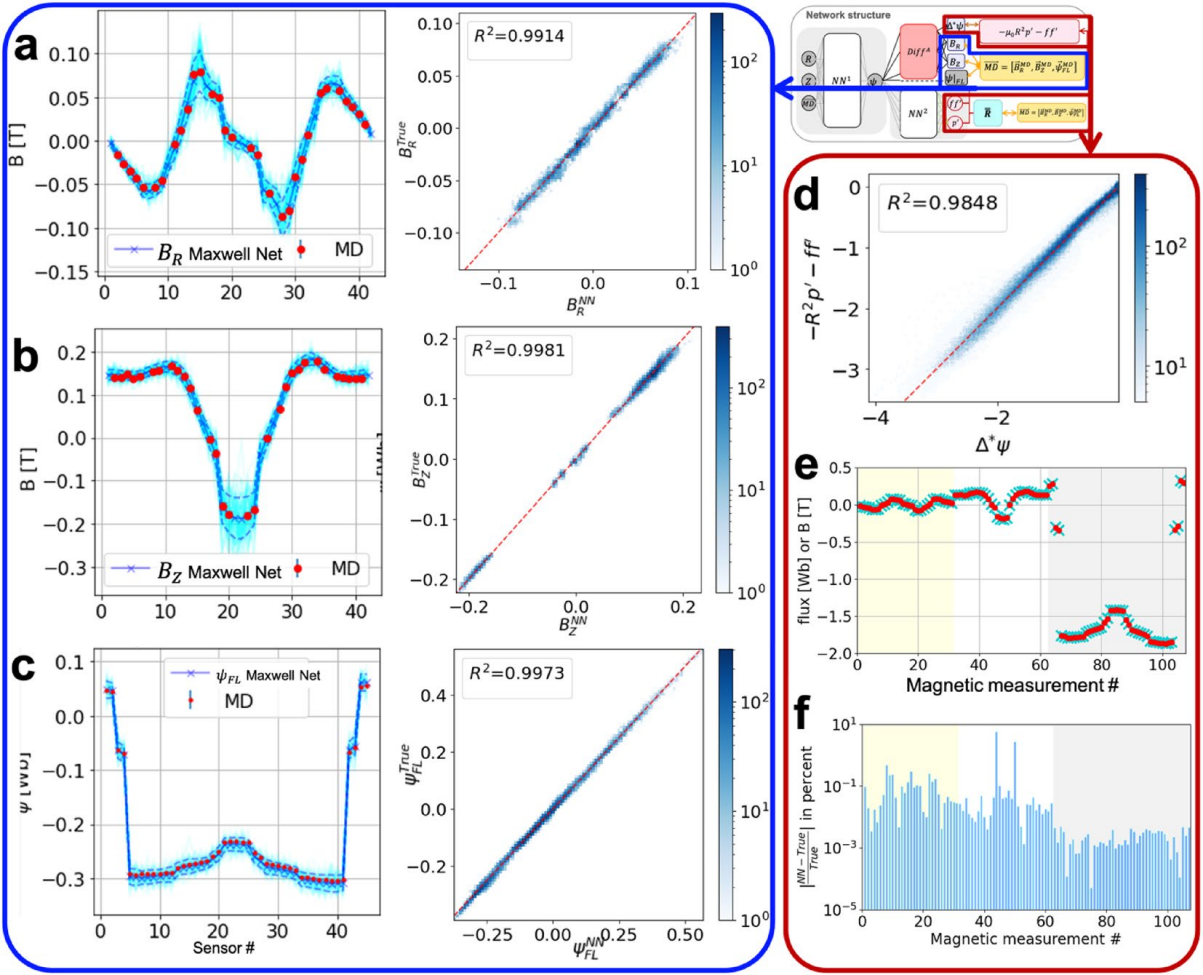
Statistical evaluation of GS-DeepNet training. (**a**–**c**) Left: profiles of (**a**) ${B}_{R}$, (**b**) ${B}_{Z}$ and (**c**) $\psi$ from the Maxwell Net (cyan) and their corresponding measurements (red) as a function of the sensor number. Cyan dashed lines represent 1 − $\sigma$ uncertainties of the network estimated from the MC dropout. Right: training statistics of the Maxwell Net, where the abscissa is the network’s output and the ordinate is the measurement for (**a**) ${B}_{R}$, (**b**) ${B}_{Z}$ and (**c**) $\psi$. The blue gradient indicates the number of occurrences and the red dashed lines are the $y=x$ lines. The coefficients of determination ${R}^{2}$ are also given. (**d**) Training statistics of the Maxwell Net (abscissa) and the Force-Balance Net (ordinate). The blue gradient indicates the number of occurrences and the red dashed line is the $y=x$ line. The coefficient of determination ${R}^{2}$ is also given. (**e**) Comparisons between ${B}_{R}$, ${B}_{Z}$ and $\psi$ (cyan crosses) calculated using the response matrix $\overline{\overline{\mathfrak{R}}}$ together with the output of the Force-Balance Net and the measurements (red circles) and (**f**) their relative errors indicated by the blue bars. In (**e**,**f**), shadings indicate different types of measurement; i.e., ${B}_{R}$ (yellow), ${B}_{Z}$ (white) and $\psi$ (gray).

GS-DeepNet is constrained only by the GS equation given as Eq. (1), which is formulated as the loss functions ${l}_{1}$ and ${l}_{2}$ (see “Methods”) as in Eqs. (6) and (7) for the Maxwell Net $N{N}_{\Theta }^{1}$ and Force-Balance Net $N{N}_{\theta }^{2}$, respectively, and by the measured magnetic signals as the boundary conditions. Therefore, we first evaluate the training performance; i.e., we evaluate how well GS-DeepNet has learned to solve the GS equation with the boundary conditions. This is important because GS-DeepNet does not rely on existing traditional numerical algorithms that solve the GS equation. Figure 2 summarizes the statistical results of the training performance.

Figure 2a–c shows the performance of the Maxwell Net in outputting values of $\psi$ and their corresponding ${\left.\left({B}_{R},{B}_{Z},\psi \right)\right|}_{MD}$ calculated by the automatic differential operator^34^ according to the boundary conditions, which are magnetic measurements $\overrightarrow{MD}=\left({\vec{B}}_{R}^{MD}, {\vec{B}}_{Z}^{MD},{\vec{\psi }}_{FL}^{MD}\right)$. Taking a single feature as an example, the left panels in Fig. 2a–c compare the GS-DeepNet results (cyan line: average; cyan dashed line: 1 − $\sigma$ uncertainty) and magnetic measurements (red dots) for ${B}_{R}$ (Fig. 2a), ${B}_{Z}$ (Fig. 2b) and ${\psi }_{FL}$ (Fig. 2c). Note that the 1 − $\sigma$ uncertainty of the network is obtained using the Monte Carlo (MC) dropout method^39^. The right panels in Fig. 2a–c show histograms (color coded), generated using all training data sets, comparing GS-DeepNet results (abscissa) and measurements (ordinate) with the $y=x$ red dashed line. We also provide values of the coefficient of determination ${R}^{2}$ for ${B}_{R}$, ${B}_{Z}$ and ${\psi }_{FL}$, all of which are close to unity, indicating that the Maxwell Net has been trained well to be consistent with the measured boundary conditions.

We mention that KSTAR has 42 magnetic pick-up probes for measuring both ${B}_{R}$ and ${B}_{Z}$ at the toroidal location that we are considering and 45 flux loops for measuring ${\psi }_{FL}$, as shown by the probe and loop indices on the horizontal axes of Fig. 2a–c. Among them, we used 31 magnetic pick-up probes because the others were impaired and simply output null values; thus, there are 31 red dots, instead of 42, in Fig. 2a and b. For the signals from the 45 flux loops, we devised an algorithm using a separate neural network that examines whether the measurements of ${\vec{\psi }}_{FL}^{MD}$ are consistent with the measurements made by the magnetic pick-up probes and, if necessary, corrects the flux-loop measurements in real time^40^; thus, we are able to use 45 measurements for ${\psi }_{FL}$ as in Fig. 2c. Note that such real-time corrections are necessary because a number of the magnetic measurements are sometimes impaired as the pick-up probes and flux loops are susceptible to damage^41,42^. One advantage of our GS-DeepNet over traditional numerical algorithms solving the GS equation is that GS-DeepNet allows us to use every magnetic measurement except those that are fully out of order. On the other hand, traditional ones require human selection of the measurements, which introduces human subjectivity, even among seemingly good measurements to guarantee numerical convergence, i.e., minimizing a chi-square estimation^12^, and to avoid obtaining unphysical plasma equilibria. Moreover, if there are flawed magnetic pick-up probes among the 31 selected probes, GS-DeepNet can incorporate an existing real-time imputation scheme^41^ based on Bayesian inference^43^ and a Gaussian process^44^.

To examine the training performance of the Force-Balance Net, we first compare an algebraic combination of the output, namely $-{\mu }_{0}{R}^{2}{p}{^\prime}-f{f}{^\prime}$, with ${\Delta }^{*}\psi$ obtained from the Maxwell Net. As dictated by the GS equation given in Eq. (1), GS-DeepNet must satisfy ${\Delta }^{*}\psi =-{\mu }_{0}{R}^{2}{p}{^\prime}-f{f}{^\prime}$ if it is well trained. Figure 2d is a histogram (color coded) generated using whole training data sets, which compares results from the Force-Balance Net (ordinate) and the Maxwell Net (abscissa) with the $y=x$ red dashed line. We see that the coefficient of determination ${R}^{2}$ is close to unity, indicating that GS-DeepNet has taught itself well to solve the GS equation.

Being free functions, $p{^\prime}(\psi )$ and $f{f}{^\prime}\left(\psi \right)$ must not only obey the GS equation as demonstrated in Fig. 2d but also comply with the magnetic measurements. Otherwise, these two free functions would have too much freedom to be applicable for tokamak operations. Figure 2e–f shows how these free functions are consistent with the magnetic measurements by comparing the Force-Balance Net results $\overline{\overline{\mathfrak{R}}} \vec{J}$ with the given magnetic data $\overrightarrow{MD}$, with the background colors of yellow, white and gray indicating ${B}_{R}$, ${B}_{Z}$ and $\psi$, respectively. Here, $\overline{\overline{\mathfrak{R}}}$ is the response matrix, and $\vec{J}$ is the current source containing toroidal current densities of plasma, and currents flowing through the external coils and the vacuum vessel (see “Methods”).

### Physical parameters learned by GS-DeepNet

We confirm that with high degrees of precision GS-DeepNet learns the GS equation by itself with the measurement constraints as the boundary conditions, and therefore we present the physical parameters that GS-DeepNet obtains—the poloidal flux function $\psi$, plasma pressure $p$ and poloidal current function $f$—to demonstrate the real-time applicability of GS-DeepNet to tokamak operations.

**Figure 3:**
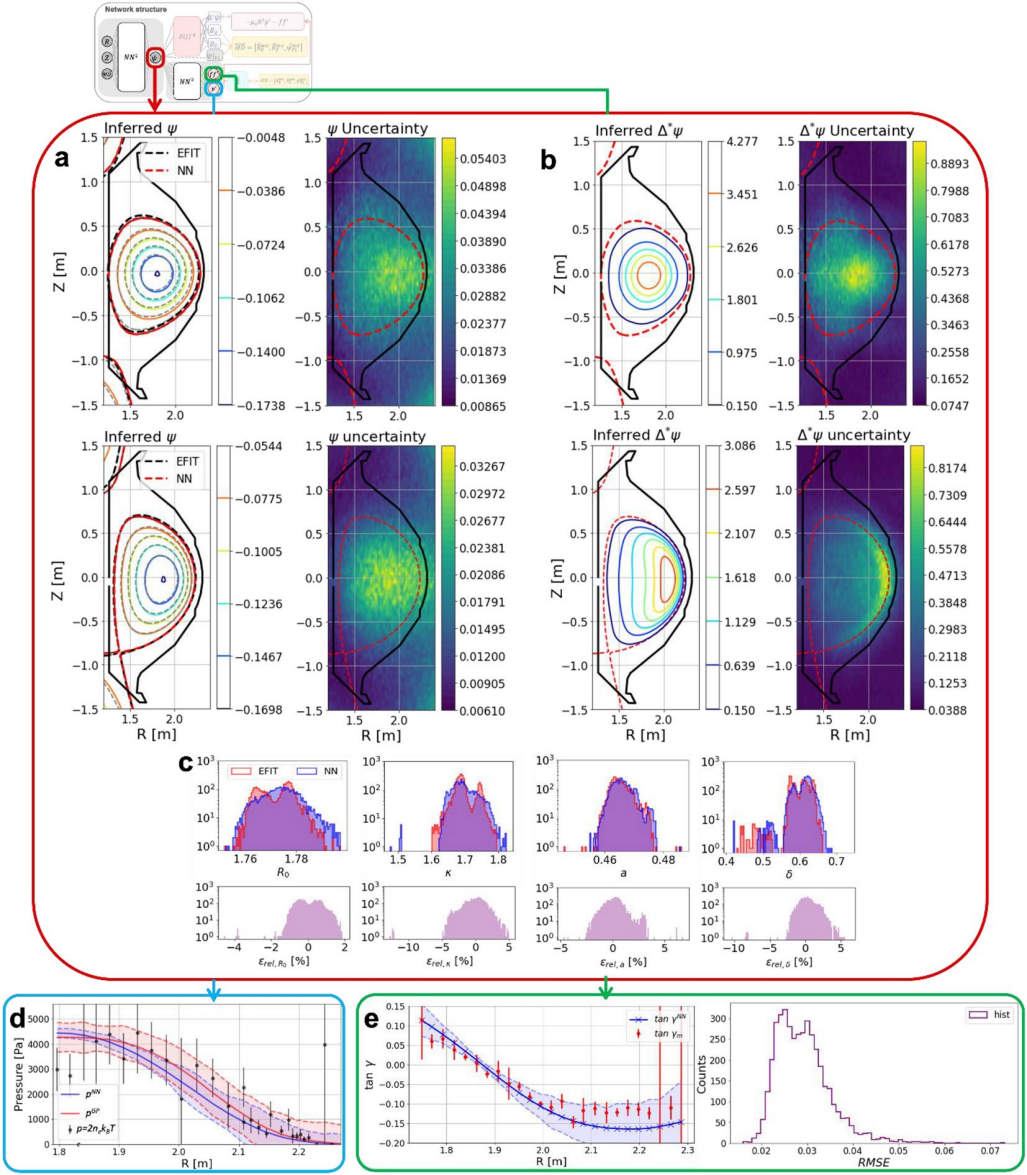
Equilibrium prediction using the trained GS-DeepNet. (**a**) First column: example of limited (top) and diverted (bottom) plasma equilibria $\psi (R, Z)$ produced by the Maxwell Net (colored lines) compared with the equilibria from EFIT (gray dashed lines). The red and black dashed lines represent the plasma boundaries from the Maxwell Net and EFIT, respectively. Second column: uncertainty maps of the Maxwell Net’s $\psi (R, Z)$. The red dashed lines indicate the last closed flux surface. (**b**) Same as in (a) for ${\Delta }^{*}\psi$ from the Maxwell Net. (**c**) Histograms of the various plasma parameters (${R}_{0}$, $\kappa$, $a$ and $\delta$) from GS-DeepNet (blue) and the EFIT (red) and their relative errors ${\epsilon }_{rel}$. (**d**) Comparisons of the profile of plasma pressure from the Force-Balance Net (blue) and the measurements (black) based on $p=2{n}_{e}{k}_{B}{T}_{e}$. The red line is obtained vis the Gaussian process from the measurements. Blue and red highlighted regions indicate the 1 − $\sigma$ uncertainty from the Force-Balance Net and Gaussian process, respectively. (**e**) Left: comparison of the profile of the calculated magnetic pitch angle, $\mathrm{tan}\gamma$, from the Force-Balance Net (blue) and the measurements (red). Right: histogram of the root-mean-square difference in $\mathrm{tan}\gamma$ between the Force-Balance Net and measurements.

Tokamak plasmas are generally categorized as limited and diverted plasmas^45^. These two types of plasma have fundamentally different magnetic topologies in that a limited plasma has its boundary touching a solid wall (Fig. 3a,b, top row), whereas a diverted plasma has one (or more) magnetic X-point(s) (i.e., null point(s) of poloidal magnetic fields) and only the legs extend to the divertors (Fig. 3a,b, bottom row). As shown in Fig. 3a,b, these two types of tokamak plasma are well reconstructed by GS-DeepNet in accordance with the measurements. Here, with example features randomly selected from KSTAR discharges, Fig. 3a shows the poloidal flux function $\psi$, which is the most important quantity with which to control the plasmas, and their corresponding ${\Delta }^{*}\psi$, which dictates the toroidal current density, is shown in Fig. 3b. Because there are absolutely no means by which to determine an equilibrium in tokamaks directly, Fig. 3a compares the contours of constant $\psi$ from an existing numerical algorithm EFIT (gray dashed lines) with those from GS-DeepNet (colored lines). Not only do the contours match well but also the plasma boundary (red dashed line) coincides with that from EFIT (black dashed line). We cannot argue whether or not the contours of $\psi$ from EFIT is true representation of the plasma equilibrium because EFIT solves the ill-posed GS equation. Nevertheless, EFIT is a reasonable target for comparison because it has been widely used in the field of nuclear fusion. Figure 3a,b also shows the model uncertainties obtained using the MC dropout method^39^.

For a complete statistical comparison, we estimated and compared histograms of several plasma parameters widely used in plasma control, namely the major radius ${R}_{0}$ ($R$ at the magnetic axis of an equilibrium), elongation $\kappa$ (the ratio of vertical to horizontal sizes of a plasma), minor radius $a$ (the half distance between the innermost and outermost $R$ positions of a plasma) and triangularity $\delta$ (a shaping parameter) (see Fig. S5g in Supplementary Note S2). Histograms for EFIT and GS-DeepNet are shown in Fig. 3c together with the differences between them calculated as ${\epsilon }_{rel,x}=100\times \left({x}^{EFIT}-{x}^{NN}\right)/{x}^{EFIT}$, where ${x}^{EFIT}$ and ${x}^{NN}$ take one of the plasma parameters from EFIT and GS-DeepNet, respectively. Although the estimations are similar between the two, noticeable differences can be seen in that the only EFIT results have the bimodal histograms for ${R}_{0}$ and $\kappa$. Whether such results are real phenomena or not cannot be identified at this point since we find that there exist at least 5 to 10 mm uncertainties in EFIT for ${R}_{0}$ and the boundary estimations, which are associated with selecting slightly (about 10%) different combinations of working magnetic pick-up coils (similar to the human subjectivity discussed earlier) as well as a finite spatial resolution the EFIT has, i.e., radial resolution of approximately 10 mm. We note that GS-DeepNet gets a position of ($R, Z$) as its input, meaning that GS-DeepNet can have a higher spatial resolution around the magnetic axis, X-point(s) and the boundary.

While one may argue that the availability of a real-time map of $\psi (R, Z)$ from the Maxwell Net of GS-DeepNet suffices for the purpose of tokamak operation, we further examine a radial profile of the plasma pressure because it is a part of the Force-Balance Net that is essential for training GS-DeepNet. Comparing the plasma pressure from GS-DeepNet with a measurement is not trivial as tokamak plasmas consist of electrons, main ions (usually isotopes of hydrogen), fast ions and trace amounts of impurity ions, such as carbon and nitrogen ions. In most cases, we can ignore the pressure contributions from the impurity ions because their densities are much lower than the density of the main ions. Thus, denoting the density and temperature by $n$ and $T$ with a subscript $e,$ $i$ and $F$ for the electron, main ion and fast ion, respectively, we have the total pressure $p={n}_{e}{k}_{B}{T}_{e}+{n}_{i}{k}_{B}{T}_{i}+{n}_{F}{k}_{B}{T}_{F}$ with the Boltzmann constant ${k}_{B}$. During tokamak operation, plasmas can be heated by their own currents with finite electrical resistivity^46^ and by injecting external electromagnetic waves^47^ resonating with either electrons or ions. There is also a neutral beam injection^48^ that heats the plasmas, which is a main source of fast ions in present tokamaks. Unfortunately, there is no method of measuring a radial pressure profile of fast ions in KSTAR; therefore, we need to select plasmas that have no fast ions (i.e., no neutral beam injections) to make the pressure comparisons with the measurements. This means that we cannot measure the pressure of main ions either because the temperature of main ions is inferred by the Charge Exchange Spectroscopy system^49^, which requires a neutral beam injection. Thus, the only available pressure measurement is that for the electrons using the Thomson scattering system^5,50^, which provides ${n}_{e}$ and ${T}_{e}$, separately. Therefore, as has been done for other tokamaks^51–53^, we simply set the measured total plasma pressure to be $p=2{n}_{e}{k}_{B}{T}_{e}$ for ohmic discharges.

Figure 3d shows a radial profile of the measured pressure as $p=2{n}_{e}{k}_{B}{T}_{e}$ (black dots) from the KSTAR Thomson scattering system^50^ for an ohmic discharge, The red line denoted as ${p}^{GP}$ is obtained by performing a non-parametric Gaussian process regression^44^ on the measurements. The pressure profile from GS-DeepNet, denoted as ${p}^{NN}$, is shown as the blue line. A comparison reveals that the pressure profile from GS-DeepNet agrees well with the measurements within their uncertainties. Note that uncertainties in ${p}^{GP}$ are obtained through the Gaussian process regression with the measurement uncertainties whereas those in ${p}^{NN}$ are estimated via the MC dropout method^39^ without relying on the measurement uncertainties. Thus, the uncertainties in ${p}^{NN}$ such as smaller uncertainties in the core compared to those in the edge require careful interpretations. This will be addressed as a future work with the extended GS-DeepNet proposed in Supplementary Note S3.

Another output of the Force-Balance Net is $${ff}{^\prime}$$, where $$f=R{B}_{\phi }$$ is the poloidal current function with the toroidal magnetic field $${B}_{\phi }$$. We examine this quantity with measured local magnetic pitch angles via the motional Stark effect (MSE) diagnostic system^6^. The MSE system in KSTAR^54^ measures the pitch angle $$\gamma$$ at 25 different radial positions as $$\mathrm{tan}\gamma \cong {A}_{1}{B}_{Z}/({A}_{2}{B}_{\phi }+{A}_{3}{B}_{R})$$, where the coefficients $${A}_{1}$$, $${A}_{2}$$ and $${A}_{3}$$ are fixed by the geometrical configuration of the system. Note that the MSE system requires a neutral beam injection, and because the pitch angle is a field quantity rather than a species quantity, we do not have to restrict ourselves only to ohmic discharges, which was the case for the pressure comparisons. Together with the output of the Maxwell Net, $$\psi$$, where we obtain $${B}_{R}(=-1/R\cdot \partial \psi /\partial Z$$$$)$$ and $${B}_{Z}(=1/R\cdot \partial \psi /\partial R)$$, $$\mathrm{tan}\gamma$$ can be calculated with the output of the Force-Balance Net. Figure 3e compares a radial profile of $$\mathrm{tan}\gamma$$, where the red dots indicate the measurements and the blue line is the result from GS-DeepNet. The agreement is excellent, and we calculated the histogram of the root-mean-square error (RMSE) of $$\mathrm{tan}\gamma$$ as $${\left\{{\sum }_{i=1}^{n}{\left(\mathrm{tan}{\gamma }_{i}^{NN}-\mathrm{tan}{\gamma }_{i}^{m}\right)}^{2}/n\right\}}^{0.5}$$ using 50 KSTAR discharges. Here, the subscript $$i$$ denotes a channel index for 25 channels (i.e., $$n=25$$), and the superscripts $$NN$$ and $$m$$ indicate values from GS-DeepNet and the measurements, respectively. As a guide for the RMSE magnitude, we note that the RMSE is 0.024 in Fig. 3e.

## Discussion

We showed that solving a two-dimensional, second-order, nonlinear, elliptic partial differential equation from scratch (i.e., *tabula rasa*) is possible with the proposed structure of an unsupervised neural network scheme. The differential equation that we solved, the GS equation, provides the most fundamental information with which to control tokamak plasmas in harnessing electricity via nuclear fusion reactions. The developed network, GS-DeepNet, comprises the Maxwell Net and Force-Balance Net and outputs the poloidal flux function $\psi$, pressure gradient $\frac{dp}{d\psi }$, and a quantity related to the poloidal current $f\frac{df}{d\psi }$ in the two-dimensional poloidal plane $(R, Z)$ consistent with the GS equation and the magnetic boundary conditions measured far from the solution space. The problem that we have solved is an ill-posed and free-boundary problem that is solved by invoking the concept of transfer learning and the singular value decomposition technique for the ill-posedness aspect and by introducing the auxiliary boundary detection module for the free-boundary aspect. Because many (if not all) physical phenomena are expressed in a form of differential equations, our work can shed light on constructing a neural network in other engineering and science fields to solve their problems starting from scratch. For instance, previous work on solving complex physical systems such as those of quantum mechanics^22,23,29^, fluid dynamics^24^ and high-energy physics^55^ may be advanced using our proposed methodologies.

Being specific to the field of nuclear fusion, GS-DeepNet is also confirmed to be capable of generating reliable magnetic equilibria when its results are compared with the results obtained from a traditional numerical algorithm, specifically EFIT. This means that GS-DeepNet can be used together with a conventional real-time EFIT algorithm to support or sometimes to rebuke real-time EFIT results because one of the advantages that GS-DeepNet has is its flexible and adjustable spatial resolution. For instance, higher spatial resolution around the magnetic axis or near the magnetic X-point(s) can be achieved with GS-DeepNet since an ($R, Z$) position is a part of its input. Therefore, using GS-DeepNet together with a real-time EFIT, we anticipate that tokamak performance will be enhanced, supporting the realization of economical nuclear fusion reactors. Note that GS-DeepNet takes less than 1 ms to estimate full two-dimensional flux surface on 41 × 41 grids using a single GPU (RTX A4000).

As a final remark, we briefly mention how GS-DeepNet can be further extended. If we know the two free functions $p(\psi )$ and $f(\psi )$, then Eq. (1) is a closed system for $\psi (R, Z)$. There thus exist advanced numerical algorithms, such as a kinetic-EFIT^56^, that consider the inferred $p(\psi )$ and $f(\psi )$ based on measurements in reconstructing the MHD equilibrium. Radial profiles of the pressure are typically measured using a Thomson scattering system^5^ for electron density and temperature, whereas the ion temperature is inferred using a charge exchange system^49^. The poloidal current function $f(\psi )$ is inferred from a profile of the local magnetic pitch angle, which can be measured using an MSE system^6^. Although there have been attempts to obtain $p(\psi )$ and $f(\psi )$ in real time^57^, these profiles are not typically available during tokamak operation. Furthermore, many of the mid- to large-scale tokamak operations contain another type of ion, namely fast ions. In some tokamak scenarios, the contribution of fast ions to the pressure is not negligible^58^, and profiles of the fast ions are not available in real time nowadays. Therefore, we do not have $p(\psi )$ and $f(\psi )$ in real time at the moment. Note that the kinetic-EFIT does not work in real time. Nevertheless, because we foresee that these profiles may become available in real time in the future, we propose an extended GS-DeepNet to accommodate such profiles. In training the Force-Balance Net with the measured $p(\psi )$ and $f(\psi )$ available in real time to output $\frac{dp}{d\psi }$ and $f\frac{df}{d\psi }$, we can add an auto-encoder as further elaborated in Supplementary Note S3.

## Methods

### Response matrix $\overline{\overline{\mathfrak{R}}}$

Let us discuss the response matrix $\overline{\overline{\mathfrak{R}}}$ in more detail because it may not be trivial. The Biot–Savart law allows us to calculate at an arbitrary spatial position the magnetic field generated using a constant electric current at a fixed location. Owing to its linearity, the magnetic field generated at a certain position by plasmas can be calculated by modeling the total toroidal plasma current as the sum of many toroidal current filaments with small rectangular cross-sections^18,59^ in a tokamak. For instance, a toroidal plasma current ${J}_{\phi }$ at a single $R-Z$ grid position (a black dot in Fig. 1b) has a well-defined rectangular cross-section set by the distance between neighboring grid points with a toroidal length of $2\pi R$ (see Fig. S2) and a fixed position. We thus pre-calculate the contribution of a single toroidal current filament ${J}_{\phi }$ generating a magnetic field at a measurement position as ${\mathfrak{R}}_{ij}$, where the subscripts $i$ and $j$ indicate a magnetic sensor and a toroidal current filament dictating the measurement and source positions, respectively. We calculate a component of the magnetic field at the location of the ${i}^{th}$ magnetic sensor due to the total toroidal plasma current as $\sum_{j}{\mathfrak{R}}_{ij}{J}_{\phi ,j}$, where $j$ runs from 1 to 1681 for the $41\times 41$ grid points.

We have a total of 107 magnetic measurements, and the contribution due to plasmas can thus be formulated as a 107 (rows) by 1681 (columns) matrix ${\overline{\overline{\mathfrak{R}}}}_{p}$. Because we do not know where the LCFS is in advance, we prepare the matrix to cover all $41\times 41$ grid points. Once the LCFS is inferred through the plasma boundary detection module, we reduce the size of ${\overline{\overline{\mathfrak{R}}}}_{p}$ to 107 (rows) by ${N}_{in}$ (columns), where ${N}_{in}$ is the number of the grid points inside the LCFS, and we denote the reduced matrix as ${\overline{\overline{\mathfrak{R}}}}_{p, in}$. Therefore, with ${\vec{J}}_{\phi }\equiv \{{J}_{\phi }\}$, (i.e., a column vector of ${N}_{in}$ by 1 containing the filaments of toroidal plasma current densities), ${\overline{\overline{\mathfrak{R}}}}_{p, in} {\vec{J}}_{\phi }$ provides a column vector with the magnitudes of magnetic fields at the 107 magnetic measurement positions due to the total plasma current. Because a tokamak has external current-carrying coils generating magnetic fields that confine hot plasmas (see Fig. 1d,e), we must also include such current sources that affect the magnetic fields at the measurement positions. At KSTAR^33^, a tokamak at which GS-DeepNet is trained, which is validated and tested for this work, 14 poloidal field coils and 16 bundles of two conductors are in four segments for the in-vessel coils where toroidal currents flow. The response matrix for these external coils, denoted as ${\overline{\overline{\mathfrak{R}}}}_{ext}$, is prepared with dimensions of 107 (rows) by 14 + 16 (columns), and the corresponding magnitudes of the currents ${\vec{J}}_{ext}$ are known because they are set using the controls of the tokamak. The other current source is the induced toroidal eddy currents flowing through the vacuum vessel that may not be negligible^60^, and this source is modeled with 18 current-carrying segments, following a previous approach used for KSTAR^61^. We thus have a 107 (rows) by 18 (columns) matrix ${\overline{\overline{\mathfrak{R}}}}_{VV}$ that contains the contribution of the eddy currents ${\vec{J}}_{VV}$ flowing through the vacuum vessel. Obviously, ${\vec{J}}_{VV}$ is a column vector with dimensions of 18 by 1, and the vector is inferred^62^ in the unsupervised training procedure. The network $N{N}_{\theta }^{2}$ must then comply with the condition $\overrightarrow{MD}=\overline{\overline{\mathfrak{R}}} \vec{J}$, where the response matrix $\overline{\overline{\mathfrak{R}}}$ is defined as $[{\overline{\overline{\mathfrak{R}}}}_{p,in}\:\vdots\: {\overline{\overline{\mathfrak{R}}}}_{ext}\:\vdots\: {\overline{\overline{\mathfrak{R}}}}_{VV}]$ with dimensions of 107 (rows) by ${N}_{in}+(14+16)+18$ (columns) and the column vector current source $\vec{J}$ is defined as $\left[{\vec{J}}_{\phi }, {\vec{J}}_{ext}, {\vec{J}}_{VV}\right]$ with dimensions of ${N}_{in}+(14+16)+18$ by 1.

Figure S2 shows the three-dimensional structure of a toroidal current filament with a rectangular cross section for modeling the plasma current density, external coil currents and vessel currents. Using this model, we estimate a matrix component of the response matrix ${\mathfrak{R}}_{ij}$ with the equations^63^

2 $${\mathfrak{R}}_{ij}^{({B}_{R})}=\frac{{\mu }_{0}}{2\pi }{\int }_{{Z}_{j,1}}^{{Z}_{j,2}}{\int }_{{R}_{j,1}}^{{R}_{j,2}}dRdZ\frac{{Z}_{i}-Z}{{R}_{i}}\sqrt{\frac{k}{4R{R}_{i}}}\left[-K\left(k\right)+\frac{{R}^{2}+{R}_{i}^{2}+{\left({Z}_{i}-Z\right)}^{2}}{{\left({R-R}_{i}\right)}^{2}+{\left({Z}_{i}-Z\right)}^{2}}E\left(k\right)\right],$$

3 $${\mathfrak{R}}_{ij}^{({B}_{Z})}=\frac{{\mu }_{0}}{2\pi }{\int }_{{Z}_{j,1}}^{{Z}_{j,2}}{\int }_{{R}_{j,1}}^{{R}_{j,2}}dRdZ\sqrt{\frac{k}{4R{R}_{i}}}\left[K\left(k\right)+\frac{{R}^{2}-{R}_{i}^{2}-{\left({Z}_{i}-Z\right)}^{2}}{{\left({R-R}_{i}\right)}^{2}+{\left({Z}_{i}-Z\right)}^{2}}E\left(k\right)\right]$$

and

4 $${\mathfrak{R}}_{ij}^{({\psi }_{FL})}=2{\mu }_{0}{R}_{i}{\int }_{{Z}_{j,1}}^{{Z}_{j,2}}{\int }_{{R}_{j,1}}^{{R}_{j,2}}dRdZ\sqrt{\frac{R}{{R}_{i}}}\frac{1}{\sqrt{k}}\left[\left(1-\frac{1}{2}k\right)K\left(k\right)-E\left(k\right)\right].$$

Here, ${\mathfrak{R}}_{ij}^{({B}_{R})}$, ${\mathfrak{R}}_{ij}^{({B}_{Z})}$ and ${\mathfrak{R}}_{ij}^{({\psi }_{FL})}$ denote the components for ${B}_{R}$, ${B}_{Z}$ and ${\psi }_{FL}$, respectively. The subscript $j$ identifies a current source (point $P$ in Fig. S2), whereas $i$ indicates a magnetic sensor (point $Q$ in Fig. S2). Thus, $\left({R}_{j,1},{R}_{j,2},{Z}_{j,1},{Z}_{j,2}\right)$ denotes the four vertices of the rectangular cross-section for the $j$th current source, whereas $\left({R}_{i},{Z}_{i}\right)$ is the spatial position of the $i$th magnetic sensor; i.e., a magnetic pick-up probe or flux loop. Note that we designate $1\le i\le 31$, $32\le i\le 62$ and $63\le i\le 107$ for 31 values of ${B}_{R}$, 31 values of ${B}_{Z}$ and 45 values of ${\psi }_{FL}$, respectively, resulting in a total of 107 values to be compared with the measured magnetic fields. We have $1\le j\le {41}^{2} \left(=1681\right)$ values for ${\overline{\overline{\mathfrak{R}}}}_{p}$, $1\le j\le 30 \left(=14+16\right)$ values for ${\overline{\overline{\mathfrak{R}}}}_{ext}$ and $1\le j\le 18$ values for ${\overline{\overline{\mathfrak{R}}}}_{VV}$. $K\left(k\right)$ and $E\left(k\right)$ are the complete elliptic integrals of the first and the second kinds, respectively, with the elliptic modulus $k=\frac{4R{R}_{i}}{{\left(R+{R}_{i}\right)}^{2}+{\left({Z}_{i}-Z\right)}^{2}}$:

5 $$K\left(k\right)={\int }_{0}^{\frac{\pi }{2}}d\theta \frac{1}{\sqrt{1-k{\mathit{sin}}^{2}\theta }} , E\left(k\right)={\int }_{0}^{\frac{\pi }{2}}d\theta \sqrt{1-k{\mathit{sin}}^{2}\theta } .$$

### How to train GS-DeepNet

To demonstrate the performance of the proposed architecture of GS-DeepNet, we randomly collected 50 experimental plasma discharges of KSTAR from 2019 campaign including a limited number of the ohmic phase and reasonable number of the L-mode and H-mode phases, where each discharge lasted a few tens of seconds. Supplementary Note S2 describes a typical KSTAR plasma discharge. From these discharges, we have obtained magnetic data at every 0.1 s starting approximately from 0.8 s of the discharge time from each discharge, resulting in $\sim {10}^{4}$ time slices (features) including ramp-up and flat-top phases but no ramp-down phases. With approximately ${2\times 10}^{3}$ spatial positions ($41\times 41+107$ positions exactly) we gathered approximately ${2\times 10}^{7} \left(={2\times 10}^{3}\times {10}^{4}\right)$ datasets, of which 80%, 5% and 15% were used as training, validation and test datasets, respectively. The training of GS-DeepNet via TensorFlow^64^ continued until it was terminated using the early stopping method (a regularization method that preserves generalization for unseen features)^65^, which took approximately 1 day with one GPU worker and 20 CPU cores using a mini-batch scheme. The total mini-batch size was approximately $8000$, which is 80% of $\sim {10}^{4}$ features. We note that measurement noise and signals drifts in the magnetic signals $\overrightarrow{MD}$ were compensated adopting a boxcar average scheme and the Bayesian-based drift mitigation method^15^, respectively. The boxcar average uses a time window of $1$ ms as the sampling frequency of the measurements is 10 kHz, and the equilibrium to control the plasmas is typically updated every ~ 10 ms in KSTAR.

Training GS-DeepNet starts by initializing both $N{N}_{\Theta }^{1}$ (Maxwell Net) and $N{N}_{\theta }^{2}$ (Force-Balance Net) with random parameters of ${\Theta }_{0}$ and ${\theta }_{0}$, respectively, adopting the Glorot (or Xavier) initialization^66^ scheme. At each iteration $k\ge 1$, we select a random and unseen feature $t$ from the total of $\sim 8000$ features, resulting in a batch size of ${41}^{2}+107=1788$ corresponding to the $41\times 41$ grid points and the $107$ different locations of the magnetic measurements. The solution ${\psi }^{t}=N{N}_{{\Theta }_{k-1}}^{1}\left(R, Z,{\overrightarrow{MD}}^{t}\right)$ is then generated at all $1788$ spatial positions, and with the automatic differential operator $Dif{f}^{A}$, $1788$ sets of ${\left({\psi , B}_{R}, {B}_{Z}, {\Delta }^{*}\psi \right)}_{t}$ are prepared. After an LCFS is inferred using the plasma boundary detection module, ${\left({p}{^\prime}, f{f}{^\prime}\right)}_{t}=N{N}_{{\theta }_{k-1}}^{2}\left({\psi }_{in}^{t}\right)$ is generated at all the positions inside the LCFS, giving us ${N}_{in}$ sets of ${\left({p}{^\prime}, f{f}{^\prime}, -{\mu }_{0}{R}^{2}{p}{^\prime}-f{f}{^\prime}, {\vec{J}}_{\phi }\right)}_{t}$. As a reminder, ${N}_{in}$ is the number of the grid points inside the LCFS. To train GS-DeepNet, we need $\vec{J}$ , which is $\left[{\vec{J}}_{\phi }, {\vec{J}}_{ext}, {\vec{J}}_{VV}\right]$; thus, we also prepare ${\vec{J}}_{ext}$ , which can be simply read from the tokamak controls, and ${\vec{J}}_{VV}$, which is determined through singular value decomposition (SVD) during the training of the Force-Balance Net. At every iteration with a randomly selected feature $t$, we update the networks’ parameters $\Theta$ and $\theta$ using the two loss functions ${l}_{1}$ and ${l}_{2}$, respectively. Following a mini-batch training scheme, one epoch is completed when GS-DeepNet sees a total of $\sim 8000$ features, corresponding to $\sim 8000$ iterations for one epoch.

We use a stochastic gradient descent algorithm to train the parameters $\Theta$ and $\theta$ by means of loss functions and the SVD technique for $\theta$ as well as $\vec{J}$. The Maxwell Net, $N{N}_{\Theta }^{1}$ together with $Dif{f}^{A}$, is trained to output an absolute value (i.e., unnormalized value) of $\psi$ such that it satisfies the boundary conditions as well as the force-balanced equilibrium. This is formulated as the loss function ${l}_{1}$:

6 $${l}_{1}={\langle {\left\{{\left.\left({B}_{R},{B}_{Z},\psi \right)\right|}_{MD}-\overrightarrow{MD}\right\}}^{2}\rangle }_{{N}_{MD}}+{\langle {\left({\Delta }^{*}{\psi }_{in}+{\mu }_{0}{R}^{2}{p}{^\prime}+{ff}{^\prime}\right)}^{2}\rangle }_{{N}_{in}}+{\langle {\left({\Delta }^{*}{\psi }_{out}\right)}^{2}\rangle }_{41\times 41-{N}_{in}}+{c}_{1}{\Vert \Theta \Vert }_{2},$$

where the angle brackets ${\langle \rangle }_{N}$ denote an averaging operator with the subscript $N$ denoting the number of elements for the average. ${\left.\left({B}_{R},{B}_{Z},\psi \right)\right|}_{MD}$ denotes the calculated ${B}_{R},{B}_{Z}$ from $Dif{f}^{A}$ and $\psi$ (output of $N{N}_{\Theta }^{1}$) at the locations of the magnetic diagnostics with ${N}_{MD}=107$; thus, the first term forces the Maxwell Net to comply with the measured boundary conditions $\overrightarrow{MD}$. The second term ensures consistency between the Maxwell Net and Force-Balance Net inside the LCFS, where ${\Delta }^{*}{\psi }_{in}$ is the estimate from the Maxwell Net and ${\mu }_{0}{R}^{2}p{^\prime}+f{f}{^\prime}$ that from the Force-Balance Net. The third term ensures that there is no toroidal plasma current outside the LCFS; i.e., ${\Delta }^{*}{\psi }_{out}=0$. The last term, ${c}_{1}{\Vert \Theta \Vert }_{2}$, is the L2 regularization (sum of squares) on the weights with the coefficient ${c}_{1}={10}^{-3}$, and it avoids overfitting together with the early stopping method^65^.

The Force-Balance Net, $N{N}_{\uptheta }^{2}$, is trained to output $\left({p}{^\prime},{ff}{^\prime}\right)$ with an input $\psi$ from the Maxwell Net after normalizing $\psi$ such that its value becomes 0 and 1 at the magnetic axis and boundary, respectively. The loss function ${l}_{2}$ for the Force-Balance Net is constructed as

7 $${l}_{2}={\left(\overline{\overline{\mathfrak{R}}} \vec{J} -\overrightarrow{MD}\right)}^{2}+{\left\{{\mathrm{\alpha }}^{*}\sum_{i=1}^{{N}_{in}}\left({R}_{i}{p}_{i}{^\prime}+\frac{{ff}_{i}{^\prime}}{{R}_{i}{\mu }_{0}}\right)-{I}_{m}^{plasma}\right\}}^{2}+{c}_{2}{\Vert \theta \Vert }_{1}.$$

The first term specifies the network to be consistent with the measured boundary conditions $\overrightarrow{MD}$ because the response matrix $\overline{\overline{\mathfrak{R}}}$ associates the current source $\vec{J}$, which is a function of the network’s output $\left({p}{^\prime},{ff}{^\prime}\right)$, with $\overrightarrow{MD}$. The second term dictates that the sum of filaments of the toroidal current density inside the LCFS calculated by the network, which is ${\mathrm{\alpha }}^{*}{\Sigma }_{\mathrm{i}=1}^{{N}_{in}}\left({R}_{i}{p}_{i}{^\prime}+{ff}_{i}{^\prime}/{R}_{i}{\mu }_{0}\right)$ with the area of the rectangular cross-section $\alpha$ divided by ${10}^{6}$ (${\mathrm{\alpha }}^{*}\equiv \alpha /{10}^{6}$), must be equal to the measured total plasma current ${I}_{m}^{plasma}$ in units of megaamperes, which is the reason why we have a factor of ${10}^{6}$ for ${\alpha }^{*}$. To regularize the weights connecting the last hidden layer and the output layer, we have the L1 regularization (sum of absolute values), ${c}_{2}{\Vert \theta \Vert }_{1}$, with the coefficient ${c}_{2}={10}^{-2}$ for $p{^\prime}$ and ${10}^{-3}$ for $ff{^\prime}$. In resolving the ill-posedness of our system, training the Force-Balance Net becomes complex as we use the concept of transfer learning^36^ and the SVD technique in addition to a usual gradient descent algorithm, which makes our algorithm novel; thus, we provide a detailed explanation of our algorithm in ‘*Optimizing the Force-Balance Net*’ in Methods.

### Optimizing the Force-Balance Net

As mentioned in the main text, the Force-Balance Net accepts a value of $\psi$ as the input from the Maxwell Net and outputs (${p}{^\prime}, ff{^\prime}$). The Force-Balance Net has two fully connected hidden layers, with the first and second layers having 60 and 6 nodes, respectively, without any bias nodes. A neural network can sensibly reproduce any form of a profile, similar to the case for Gaussian processes, as long as the network has a sufficient number of degrees of freedom^67^. We find empirically that our choice of the numbers of hidden layers and nodes has enough degrees of freedom to describe the experimentally measured profiles of ${p}{^\prime}$ and $f{f}{^\prime}$.

Optimizing the Force-Balance Net comes down to a problem of minimizing the loss function ${l}_{2}$ (Eq. (7)), whose role is to reveal the most probable representations of $\vec{J}=\left[{\vec{J}}_{\phi }, {\vec{J}}_{ext}, {\vec{J}}_{VV}\right]$ that explain the given magnetic data $\overrightarrow{MD}$ and the measured total plasma current ${I}_{m}^{plasma}$ with consideration of the L1 regularization, where ${\vec{J}}_{\phi }$ is determined by an algebraic combination of ${\vec{p}}{^\prime}$ and ${\vec{ff}}{^\prime}$. As a reminder, ${\vec{J}}_{\phi }$ is the collection of filaments of the toroidal plasma currents, whereas ${\vec{J}}_{ext}$ and ${\vec{J}}_{VV}$ are the currents flowing through the external magnetic field coils and the eddy currents flowing through the vacuum vessel, respectively. This optimization process is generally subject to an ill-posed condition because the number of unknown quantities to be determined (i.e., ${\vec{p}}{^\prime}$, ${\vec{ff}}{^\prime}$ and ${\vec{J}}_{VV}$) is considerably larger than the number of the given measurements (i.e., $\overrightarrow{MD}$ and ${I}_{m}^{plasma}$). Note that we have reasonably good information on ${\vec{J}}_{ext}$ because it is a control parameter. Consequently, optimizing the Force-Balance Net using only a gradient descent algorithm often leads the to-be-determined current density $\vec{J}$ to be stuck in extremely unphysical solution spaces where very small plasma currents of the order of a few amperes throughout the plasma region are predicted while a small number of segments of eddy current on the vacuum vessel attempt to explain the entire set of measurements. This is likely to have at least two reasons. First, there are many nuisance local minima owing to the ill-posed condition. Second, constraints on the eddy current ${\vec{J}}_{VV}$ are very weak. Therefore, we relax the ill-posed condition by recasting the loss function ${l}_{2}$ into a matrix multiplication form and then optimize the source current $\vec{J}=\left[{\vec{J}}_{\phi }, {\vec{J}}_{ext}, {\vec{J}}_{VV}\right]$ by updating the weights of direct connections to the output nodes using the SVD technique, while all other weights up to the last hidden layer are determined using a gradient descent algorithm through transfer learning^36^.

Figure S3a shows the matrix form of the loss function ${l}_{2}$. We introduce a clever representation for the plasma current density ${\vec{J}}_{\phi }$ and its associated response matrix ${\overline{\overline{\mathfrak{R}}}}_{p, in}$ to loosen the ill-posed condition, while ${\vec{J}}_{ext}$ and ${\vec{J}}_{VV}$ and their associated response matrices ${\overline{\overline{\mathfrak{R}}}}_{ext}$ and ${\overline{\overline{\mathfrak{R}}}}_{VV}$ are expressed as they are (the red part in Fig. S3a). Note that we borrow the idea of the matrix representation from the EFIT algorithm^12^. These quantities regulate the first term of the loss function ${l}_{2}$ as we wish to minimize ${\left(\overline{\overline{\mathfrak{R}}} \vec{J} -\overrightarrow{MD}\right)}^{2}$. Basically, we reform the plasma contributions, namely ${\overline{\overline{\mathfrak{R}}}}_{p,in}{\vec{J}}_{\phi }$ and ${\mathrm{\alpha }}^{*}{\Sigma }_{\mathrm{i}=1}^{{N}_{in}}\left({R}_{i}{p}_{i}{^\prime}+{ff}_{i}{^\prime}/{R}_{i}{\mu }_{0}\right)$, in terms of the network’s weight-wise form as ${\overline{\overline{\mathfrak{R}}}}_{p,in}{\bar{\bar{h}}}^{\star }{\vec{v}}_{{\vec{J}}_{\phi }}$ and ${\vec{\alpha }}^{*}{\bar{\bar{h}}}^{\star }{\vec{v}}_{{\vec{J}}_{\phi }}$ (see the red part in Fig. S3a), respectively, with ${\bar{\bar{h}}}^{\star }{\vec{v}}_{{\vec{J}}_{\phi }}={\vec{J}}_{\phi }$ (see Fig. S3b), where ${\bar{\bar{h}}}^{\star }$ and ${\vec{v}}_{{\vec{J}}_{\phi }}$ are explained below. Let us suppose that we want to estimate a plasma current density ${J}_{\phi ,i}$ at the ${i}^{th}$ grid position inside the LCFS with an output of the Force-Balance Net $({p}_{i}{^\prime}, f{f}_{i}{^\prime})$. We know that ${J}_{\phi ,i}={R}_{i}{p}_{i}{^\prime}+f{f}_{i}{^\prime}/{R}_{i}{\mu }_{0}$ from Eq. (1), and ${p}_{i}{^\prime}={\vec{h}}_{i}{\vec{v}}_{{p}{^\prime}}$ and ${ff}_{i}{^\prime}={\vec{h}}_{i}{\vec{v}}_{f{f}{^\prime}}$ from the network’s structure with the node values at the last hidden layer ${\vec{h}}_{i}$ (dimensions of $1\times 6$) and the weights assigned to the output from the last hidden layer ${\vec{v}}_{{p}{^\prime}}$ (dimensions of $6\times 1$) and ${\vec{v}}_{f{f}{^\prime}}$ (dimensions of $6\times 1$) for the output nodes ${p}{^\prime}$ and $f{f}{^\prime}$, respectively. We thus rewrite ${J}_{\phi ,i}$ in the weight-wise form as ${J}_{\phi ,i}={R}_{i}{\vec{h}}_{i}{\vec{v}}_{{p}{^\prime}}+\frac{1}{{R}_{i}{\mu }_{0}}{\vec{h}}_{i}{\vec{v}}_{f{f}{^\prime}}$. This weight-wise representation can be extended to all grid positions inside the LCFS as ${\vec{p}}{^\prime}=\bar{\bar{h}}{\vec{v}}_{{p}{^\prime}}$ and ${\vec{ff}}{^\prime}=\bar{\bar{h}}{\vec{v}}_{f{f}{^\prime}}$ with the matrix form of the hidden node values $\bar{\bar{h}}$ (dimensions of ${N}_{in}\times 6$). Let us define two constant matrices containing ${R}_{i}$ and $\frac{1}{{R}_{i}{\mu }_{0}}$ as ${\overline{\overline{\mathfrak{C}}}}^{p{^\prime}}$ and ${\overline{\overline{\mathfrak{C}}}}^{ff{^\prime}}$ such that ${\overline{\overline{\mathfrak{C}}}}^{p{^\prime}}=\left[{\mathfrak{C}}_{ij}^{{p}{^\prime}}\right]={R}_{i}$ and ${\overline{\overline{\mathfrak{C}}}}^{ff{^\prime}}=\left[{\mathfrak{C}}_{ij}^{{ff}{^\prime}}\right]=\frac{1}{{R}_{i}{\mu }_{0}}$, whose dimensions are ${N}_{in}\times 6$ each. We then have ${\vec{J}}_{\phi }={\bar{\bar{h}}}^{\star }{\vec{v}}_{{\vec{J}}_{\phi }}$, where ${\bar{\bar{h}}}^{\star }$ is a concatenated (or augmented) matrix $\left[ {\overline{{\overline{{\mathfrak{C}}} }}^{p\prime } \odot \bar{\bar{h}} \:\vdots \:\overline{{\overline{{\mathfrak{C}}} }}^{ff\prime } \odot \bar{\bar{h}}} \right]$ (dimensions of ${N}_{in}\times 12$) with the Hadamard product $( \odot )$, and ${\vec{v}}_{{\vec{J}}_{\phi }}=[{\vec{v}}_{{p}{^\prime}}, {\vec{v}}_{{ff}{^\prime}}]$ (dimensions of $12\times 1$) (see Fig. S3b). We can thus change ${\overline{\overline{\mathfrak{R}}}}_{p,in}{\vec{J}}_{\phi }$ into ${\overline{\overline{\mathfrak{R}}}}_{p,in}{\bar{\bar{h}}}^{\star }{\vec{v}}_{{\vec{J}}_{\phi }}$, which is what we have in Fig. S3a. Similarly, a predicted total plasma current ${\mathrm{\alpha }}^{*}{\Sigma }_{\mathrm{i}=1}^{{N}_{in}}\left({R}_{i}{p}_{i}{^\prime}+{ff}_{i}{^\prime}/{R}_{i}{\mu }_{0}\right)$ can be represented as ${\vec{\alpha }}^{*}{\bar{\bar{h}}}^{\star }{\vec{v}}_{{\vec{J}}_{\phi }}$ using a row vector ${\vec{\alpha }}^{*}=\left[{\mathrm{\alpha }}^{*},\cdots ,{\mathrm{\alpha }}^{*}\right]$ with dimensions of $1\times {N}_{in}$, and it plays a role as the second term in the loss function ${l}_{2}$ ; i.e., minimizing ${\left\{{\mathrm{\alpha }}^{*}{\Sigma }_{\mathrm{i}=1}^{{N}_{in}}\left({R}_{i}{p}_{i}{^\prime}+{ff}_{i}{^\prime}/{R}_{i}{\mu }_{0}\right)-{I}_{m}^{plasma}\right\}}^{2}$. The key rationale behind reformulating ${\vec{J}}_{\phi }$ in the weight-wise form is that instead of letting the measured $\overrightarrow{MD}$ and ${I}_{m}^{plasma}$ be explained by ${\vec{J}}_{\phi }$ itself, which is the main cause of the ill-posed condition because ${\vec{J}}_{\phi }$ occupies the majority of $\vec{J}$, we design the given measurements to be described by ${\vec{v}}_{{\vec{J}}_{\phi }}$, which drastically reduces the number of unknown parameters and thus relaxes the ill-posed condition.

The third term in the loss function ${l}_{2}$ is the L1 regularization, and its purpose is to prevent the network’s output from being unnecessarily complex; i.e., preventing the overfitting problem. This regularization can be embedded in the matrix form; i.e., forcing ${c}_{2}\overline{\overline{I}}{\vec{v}}_{{\vec{J}}_{\phi }}$ to be as small as possible (see the blue part in Fig. S3a), where the identity matrix $\overline{\overline{I}}$ has dimensions of $12\times 12$. ${c}_{2}$ is constant, and we set it at ${10}^{-2}$ for ${\vec{v}}_{{p}{^\prime}}$ and ${10}^{-3}$ for ${\vec{v}}_{ff{^\prime}}$. Indeed, ${c}_{2}$ plays a crucial role in determining how flexible and complex the profiles of $p{^\prime}$ and $ff{^\prime}$ can be, and we determined its value using numerous profiles of $p{^\prime}$ and $ff{^\prime}$ obtained from KSTAR over the past few years such that the Force-Balance Net has just enough, and not too much, flexibility to satisfy the given measurements.

We must also consider that some of the externally generated magnetic fields have finite degrees of distortion owing to the Incoloy effect^68^. KSTAR has 14 poloidal field coils, with 10 of them being subject to the Incoloy effect as they are made of Nb_3_Sn superconductors with the Incoloy 908 conduit, whereas the other four are made of NbTi superconductors and STS316LN conduit^69^. Let us introduce ${\vec{J}}_{ext}^{m}$ to denote the actual currents flowing through the external magnetic field coils, which are always available in real time during tokamak operation. If we use ${\overline{\overline{\mathfrak{R}}}}_{ext}{\vec{J}}_{ext}^{m}$ as a part of the calculated $\overline{\overline{\mathfrak{R}}} \vec{J}$ and compare it with $\overrightarrow{MD}$, (i.e., we let ${{\vec{J}}_{ext}=\vec{J}}_{ext}^{m}$), we will generate bias errors because our ${\overline{\overline{\mathfrak{R}}}}_{ext}$ does not model the Incoloy effect. Instead of rectifying ${\overline{\overline{\mathfrak{R}}}}_{ext}$ to include such an effect, which is a demanding and challenging task, we relax the condition of ${{\vec{J}}_{ext}=\vec{J}}_{ext}^{m}$ and let the network determine ${\vec{J}}_{ext}$ that is consistent with $\overrightarrow{MD}$. In other words, we allow ${\vec{J}}_{ext}$ to be the effective external currents including the consequence of the Incoloy effect. This is done by introducing a diagonal matrix $\bar{\bar{\beta } }$, whose dimensions are $30\times 30$, to relate ${\vec{J}}_{ext}$ to ${\vec{J}}_{ext}^{m}$ (see the green part in Fig. S3a). Among the diagonal components ${\beta }_{n,n}$, where the subscript $n$ indicates one of the 30 external magnetic field coils, we set ${\beta }_{n,n}=0.9$ for the external coils susceptible to the Incoloy effect (i.e., the 10 poloidal field coils), whereas the others are set to be unity. ${\vec{J}}_{ext}^{m}$ is also multiplied by either 0.9 or 1, accordingly. Setting ${\beta }_{n,n}$ less than unity essentially means that a smaller contribution to the ${\chi }^{2}$ minimization in solving the matrix using the SVD technique is allowed as we have recast the problem to be overdetermined. Note that the value of 0.9 is also used in the KSTAR EFIT algorithm.

Our matrix form of the loss function ${l}_{2}$ can also include other constraints such as the Dirichlet and/or Neumann boundary conditions. As an example, the Dirichlet boundary condition can be included such that the Force-Balance Net’s output $({p}{^\prime}, f{f}{^\prime})$ must equal zero outside the plasma-facing component (see the black part in Fig. S3a); i.e., ${\vec{h}}_{pfc}{\vec{v}}_{{p}{^\prime}}=0$ and ${\vec{h}}_{pfc}{\vec{v}}_{f{f}{^\prime}}=0$, where ${\vec{h}}_{pfc}$ (dimensions of $1\times 6$) contains the node values at the last hidden layer outside the plasma-facing component.

We now have a complete matrix form of the loss function ${l}_{2}$ in Eq. (7), which resolves the ill-posed problem by expressing the unknown ${\vec{J}}_{\phi }$ in terms of the network’s weight-wise form  ${\vec{v}}_{{\vec{J}}_{\phi }}$ and modifying the response matrix $\overline{\overline{\mathfrak{R}}}$ to contain the node values of the last hidden layer. All the necessary constraints associated with the loss function ${l}_{2}$ are modeled in the matrix $\overline{\overline{\mathcal{M}}}$ (see Fig. S3a). The unknown current sources $\vec{J}=\left[{\vec{J}}_{\phi }, {\vec{J}}_{ext}, {\vec{J}}_{VV}\right]$ have become ${\vec{J}}^{*}=\left[{\vec{v}}_{{\vec{J}}_{\phi }}, {\vec{J}}_{ext}, {\vec{J}}_{VV}\right]$, and with the real-time available measurements $\vec{g}$, we have $\vec{g}=\overline{\overline{\mathcal{M}}}{\overrightarrow{J}}^{*}$ (see Fig. S3a). This overdetermined problem can be readily solved using the SVD technique to obtain the unique ${\vec{J}}^{*}=\left[{\vec{v}}_{{\vec{J}}_{\phi }}, {\vec{J}}_{ext}, {\vec{J}}_{VV}\right]$ whose ${\chi }^{2}$ is a minimum.

As the matrix form, we have generated only updates of the weights of connection from the last hidden layer to the output layer (i.e., ${\vec{v}}_{{\vec{J}}_{\phi }}$,), and we need a way to update the remaining weights from the input layer up to the last hidden layer. This is where we use the transfer learning scheme in the Force-Balance Net. The basic idea of transfer learning is that a network trained with certain data sets (e.g., images of cats) can be transferred to other similar data sets (e.g., images of dogs). For instance, all the weights of a network pretrained with images of cats are reused except the weights of connections from the last hidden layer to the output layer, and only these weights are retrained with images of dogs. This allows us to save computational resources during the training phase. Furthermore, the transfer learning scheme allows us to work with a limited volume of data. Thus, as we do not use measured ${p}{^\prime}$ and $f{f}{^\prime}$ in training the Force-Balance Net, the transfer learning scheme is a sagacious choice for pretraining the network.

The detailed features of the profiles are handled by the SVD technique described above. We thus pretrain the network with the transfer learning scheme to weakly force a sinusoidal function with three full cycles within the last closed flux surface, allowing the profiles to have a certain level of non-monotonic features. In the pretraining, we use a normalized input domain, namely ${\psi }_{norm}=\frac{\psi -{\psi }_{axis}}{{\psi }_{LCFS}-{\psi }_{axis}}$, where ${\psi }_{LCFS}$ and ${\psi }_{axis}$ are the values of $\psi$ at the last closed flux surface and the magnetic axis, respectively. The loss function ${l}_{3}$ for the pretraining is thus defined as

8 $${l}_{3}=\frac{1}{K}\sum_{i=1}^{K}{\left({y}_{i}^{NN}-{y}_{i}\right)}^{2},$$

where ${y}^{NN}$ is the output of the Force-Balance Net (i.e., ${p}{^\prime}$ or $f{f}{^\prime}$) and $y=\mathrm{sin}6\pi {\psi }_{norm}$ is the target function. Here, the subscript $i$ indicates the normalized spatial position, and *K* is the total number of data points. The network is pretrained adopting the gradient descent algorithm with a dropout rate of 0.10 and L1 regularization.

The optimization of the Force-Balance Net can be summarized as first pretraining the network to produce a general non-monotonic spatial profile, fixing all the weights except those of connections from the last hidden layer to the output layer adopting the transfer learning scheme, and updating these weights using the SVD technique, where we introduce both physics constraints (i.e., the GS equation) and boundary conditions (i.e., real-time available measurements).

### Auxiliary module for the boundary detection

The plasma boundary, known as the LCFS, separates the region inside a tokamak vacuum vessel into two regions: confined and non-confined regions. Basically, the confined region has well-defined closed magnetic flux surfaces whereas the non-confined region has open magnetic field lines that make direct contact with the walls (i.e., divertors or plasma-facing components), allowing plasma to be lost to the walls by flowing through these field lines. The main purpose of the auxiliary boundary detection module is to find where the separation of these two regions occurs, which means that the module’s task is to find the value of $\psi$ at the LCFS because the constant contour line of ${\psi }_{LCFS}$ on the $\left(R, Z\right)$ plane separates the two regions. Here, ${\psi }_{LCFS}$ is the value of $\psi$ at the LCFS.

The boundary detection module first receives $\left(\psi ,{B}_{R},{B}_{Z}\right)$ over all $41\times 41$ grid points (black dots in Fig. 1b) and the values of $\psi$ at the plasma-facing components, denoted as ${\psi }_{PFC}$, from the Maxwell Net at each iteration within an epoch. The module then searches for the three smallest values of the poloidal magnetic fields ${B}_{p}$ by calculating ${B}_{p}=\sqrt{{B}_{R}^{2}+{B}_{Z}^{2}}$ at all $41\times 41$ grid points and finds their corresponding $\left(R, Z\right)$ positions. This is motivated by the fact that a magnetic X-point, which is located on the LCFS by definition, ideally has ${B}_{p}=0$. The magnetic axis, which is the magnetic center of the confined region, also has ${B}_{p}=0$, and we thus constrain a possible region of the magnetic X-point to be $\left|Z\right|>0.5$ m according to our prior knowledge from many existing KSTAR discharges. Let us denote the values of $\psi$ at the three positions with smallest ${B}_{p}$ as ${\psi }_{{B}_{p}^{min}}^{i=\mathrm{1,2},3}$ and the values of $\psi$ with the constraint $\left|Z\right|>0.5$ m as ${\psi }_{{B}_{p}^{min}}^{\left|Z\right|>0.5}$. We then create a set, containing candidates of ${\psi }_{LCFS}$, of absolute values of $\psi$, which is ${\psi }_{LCFS}^{candidate}=\left\{\left|{\psi }_{{B}_{p}^{min}}^{\left|Z\right|>0.5}\right|, \left|{\psi }_{PFC}\right|\right\}$. Finally, we obtain ${\psi }_{LCFS}=\mathrm{sgn}\: \mathrm{max}({\psi }_{LCFS}^{candidate})$, where $\mathrm{max}( )$ operator returns the maximum value of the set, and $\mathrm{sgn}$ is the original sign ($+$ or $-$) of the maximum value. Note that if ${\psi }_{LCFS}$ is determined by ${\psi }_{PFC}$, then we have a limited plasma (Fig. 3a,b, top row); otherwise, we have a diverted plasma (Fig. 3a,b, bottom row). We note that the LCFS must be identified routinely during tokamak operation as the plasma boundary is continuously evolving (Fig. S5f), and ${\psi }_{{B}_{p}^{min}}^{i=\mathrm{1,2},3}$ must be always found because we do not know whether we have a limited or diverted plasma in advance.

The loss functions ${l}_{1}$ and ${l}_{2}$ in Eqs. (6) and (7) must be able to distinguish between inside and outside the LCFS. This can be performed with ${\psi }_{LCFS}$ because we know that the inside means $\psi <{\psi }_{LCFS}$ (note that KSTAR has $\psi <0$ in the confined region, see Fig. 3a). This is clear when the training of GS-DeepNet is finished and provides well-behaved flux surfaces. However, during the training phase, especially the early stage, we are most likely to have randomly scattered $\left(R, Z\right)$ locations that satisfy $\psi <{\psi }_{LCFS}$. Thus, to facilitate the training phase, we introduce another constraint to be inside the LCFS; i.e., the $Z$ position must be within ${Z}_{min}$ and ${Z}_{max}$, where ${Z}_{min(max)}$ is the minimum (maximum) value of $Z$ at the three positions with smallest ${B}_{p}$ that are used to determine ${\psi }_{{B}_{p}^{min}}^{i=\mathrm{1,2},3}$.

We note that the described procedure on finding a plasma boundary is specific to KSTAR plasmas. If one expects more complicated plasma boundaries, e.g., snowflake shaped boundaries, then the boundary detection algorithm must be modified accordingly. As the boundary shapes may differ for different tokamaks, we have developed the boundary detection algorithm as an auxiliary module so that modifications on the main part of GS-DeepNet for its application to other tokamaks can be minimal.

### Supplementary Information

Supplementary Information.

## Data Availability

The data that support the plots within this paper and other findings of this study are available from the corresponding author upon reasonable request.
