# Supplementary material for: GS-DeepNet: mastering tokamak plasma equilibria with deep neural networks and the Grad–Shafranov equation
Source: Sci Rep. 2023 Sep 22;13:15799. doi: 10.1038/s41598-023-42991-5 (PMC10516960; doi:10.1038/s41598-023-42991-5)
Supplement: Supplementary file 1 — Supplementary Information. [file 41598_2023_42991_MOESM1_ESM.docx]

**Supplementary information**

**GS-DeepNet: Mastering tokamak plasma equilibria with deep neural networks and the Grad–Shafranov equation**

Semin Joung,^1,5^* Y.-C. Ghim,^1^* Jaewook Kim,^2^ Sehyun Kwak,^3^ Daeho Kwon, ^4^ C. Sung,^1^ D. Kim,^1^ Hyun-Seok Kim,^2^ J.G. Bak,^2^ S.W. Yoon^2^

^1^Department of Nuclear and Quantum Engineering, KAIST, Daejeon 34141, S. Korea, ^2^Korea institute of Fusion Energy, Daejeon 34133, S. Korea, ^3^Max-Planck-Institute fur Plasmaphysik, Greifswald D-17491, Germany, ^4^Mobiis Co., Ltd., Seongnam-si, Gyeonggi-do 13486, S. Korea, ^5^University of Wisconsin–Madison, Madison, Wisconsin 53706, USA

*Correspondence to: [semin.joung@wisc.edu](mailto:semin.joung@wisc.edu); [ycghim@kaist.ac.kr](mailto:ycghim@kaist.ac.kr)

Contents

[Supplementary Figures 2](#_Toc130407044)

[Supplementary Notes 6](#_Toc130407045)

[Note S1: Effect of a boundary condition in solving a differential equation with a neural network 6](#_Toc130407046)

[Note S2: Typical KSTAR experimental discharge and its equilibrium characteristics 10](#_Toc130407047)

[Note S3: Conceptual design of GS-DeepNet with kinetic constraints 13](#_Toc130407048)

[Supplementary References 15](#_Toc130407049)

# Supplementary Figures


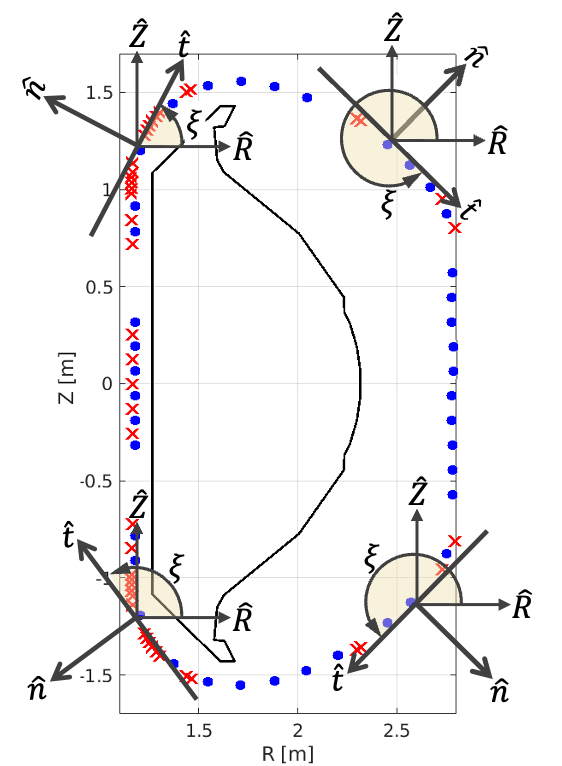


**Fig. S1.**

**Coordinate transformation for magnetic fields measured with the magnetic pick-up probes.** We convert the measured $B_{n,i}^{MD}$ and $B_{t,i}^{MD}$ into $B_{R,i}^{MD}\left( =-B_{n,i}^{MD}\sin\xi_{i}+B_{t,i}^{MD}\cos\xi_{i} \right)$ and $B_{Z,i}^{MD}\left( =B_{n,i}^{MD}\cos\xi_{i}+B_{t,i}^{MD}\sin\xi_{i} \right)$ using the angle $\xi_{i}$ between the major radius direction $\hat{R}$ and the direction tangential to the vessel wall $\hat{t}$, which is determined by the KSTAR geometry and the location of the $i$^th^ magnetic pick-up probe [1]. Here, the subscripts $n$, $t$, $R$ and $Z$ indicate the corresponding directions shown in the figure. Measurements from all the pick-up probes are transformed with this coordinate conversion scheme such that they can be used as boundary conditions for the Grad–Shafranov equation.


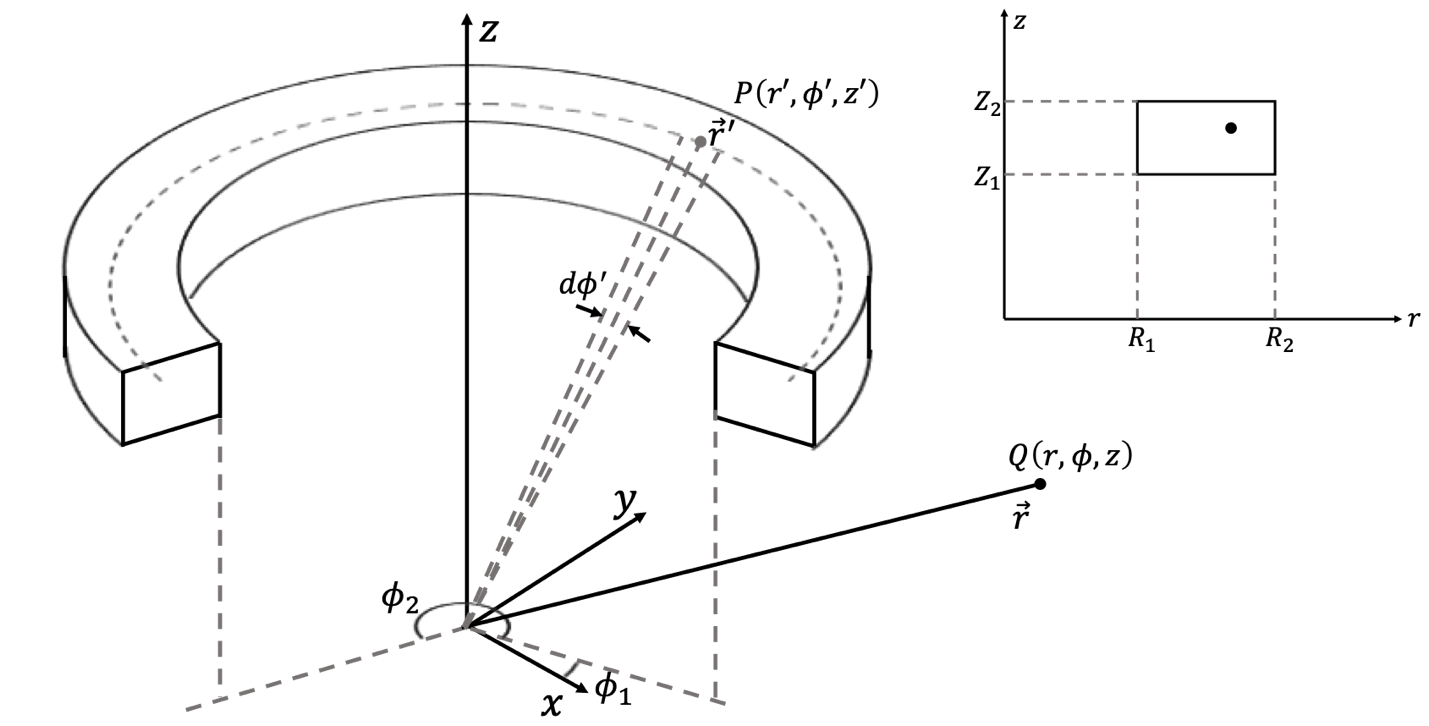


**Fig. S2.**

**Toroidal current filament with a rectangular cross section.** The coordinates $\left( R_{1},R_{2},Z_{1},Z_{2} \right)$ in the right-hand corner represent four vertices of the rectangular cross section for a toroidal current filament that is used to generate the response matrix $\mathfrak{R}$, relating the toroidal current source to the magnetic field at an arbitrary spatial position via the Biot–Savart law. The current source position $\vec{r}^{'}=P\left( r^{'},\phi^{'},z^{'} \right)$ is restricted within $R_{1}\leq r^{'}\leq R_{2}$ and $Z_{1}\leq z^{'}\leq Z_{2}$, and we denote the location of the $i^{th}$ magnetic pick-up probe as $\vec{r}=Q\left( r,\phi,z \right)$. The current filament is a toroidally closed loop, and we thus have $\left( \phi_{2}-\phi_{1} \right)=2\pi$. This figure is generated from, and thus similar to, Fig. 1 in Ref. [2].


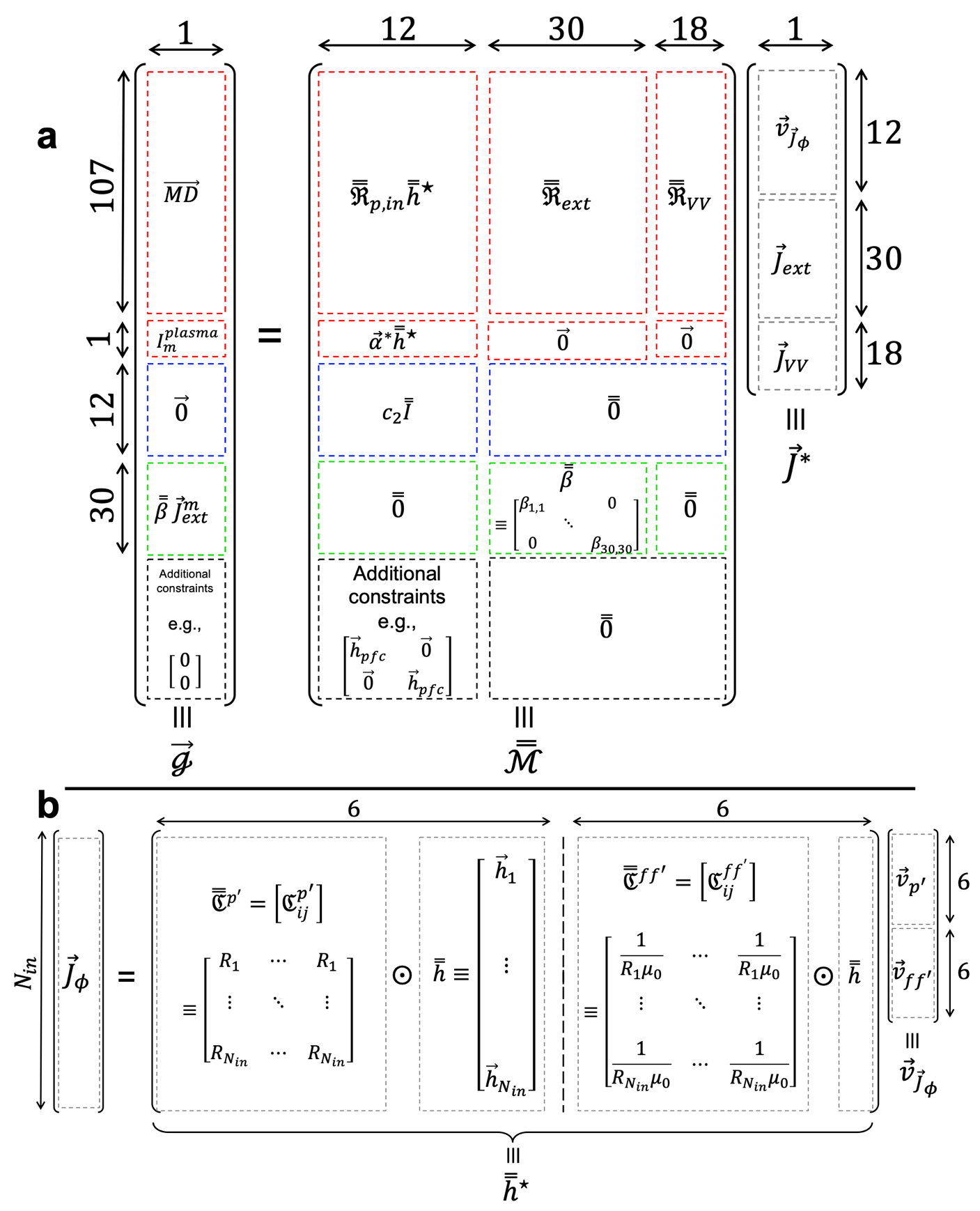


**Fig. S3.**

**Matrix representation of the loss function** $\boldsymbol{l}_{\boldsymbol{2}}$**.** The Force-Balance Net is first pretrained with the target function of a sinusoidal function. Then, only the weights connecting from the last hidden layer to the output layer are updated using the SVD technique, while the remaining weights are kept unchanged (in what is referred to as the transfer learning scheme). This figure describes how we recast the loss function $l_{2}$ into a matrix form such that the SVD techniques can be used. (a) The physical and numerical constraints and the real-time available measurements (boundary conditions) denoted as $\vec{\mathcal{g}}$ must be equal to $\mathcal{M}\vec{J}^{*}$, where $\mathcal{M}$ is the overall response matrix and $\vec{J}^{*}=\left[ \vec{v}_{\vec{J}_{\phi}}, \vec{J}_{ext}, \vec{J}_{VV} \right]$ is the unknown parameter. Note that $\vec{v}_{\vec{J}_{\phi}}$ denotes the network’s weights of connections from the last hidden layer to the output layer. The red part includes the modified response matrix $\mathfrak{R}$ constructed using the Biot–Savart law, and it dictates the first and second terms in the loss function $l_{2}$. The L1 regularization for the weights $\vec{v}_{\vec{J}_{\phi}}$ is modeled in the blue part. The green part is designed to include the Incoloy effect for $\vec{J}_{ext}$. The black part can include any other extra constraints, such as the Dirichlet and/or the Neumann boundary conditions. (b) The Grad–Shafranov equation states that we must have $J_{\phi}=Rp^{'}+\frac{1}{R\mu_{o}}ff'$. From the network’s structure, we have $p_{i}^{'}=\vec{h}_{i}\vec{v}_{p^{'}}$ and ${ff}_{i}^{'}=\vec{h}_{i}\vec{v}_{ff^{'}}$, where the subscript $i$ denotes the $i^{\text{th}}$ grid (spatial) position. $\vec{h}_{i}$ denotes the node values at the last hidden layer, and $\vec{v}_{p^{'}}$ and $\vec{v}_{ff^{'}}$ are the weights assigned to the output $p'$ and $ff'$, respectively; i.e., $\vec{v}_{\vec{J}_{\phi}}=[\vec{v}_{p^{'}}, \vec{v}_{{ff}^{'}}]$. Thus, with the Hadamard product $⨀$, we can represent the unknown plasma toroidal current density $\vec{J}_{\phi}=h^{\star}\vec{v}_{\vec{J}_{\phi}}$ as in the figure, which reduces the number of unknowns from $N_{in}$ to 12. This representation of $\vec{J}_{\phi}$ in terms of the network’s weights is used in the red part of (a).

# Supplementary Notes

## **Note S1: Effect of a boundary condition in solving a differential equation with a neural network**

Let us take the simple first-order linear differential equation

|  | $\frac{d}{dx}t(x)-14\pi\cos\left( 14\pi x \right)=0,$ | (S1) |
| --- | --- | --- |

whose solution is $t\left( x \right)=\sin\left( 14\pi x \right)+t_{0}$, where $t_{0}$ is an integration constant. We now wish to have a neural network to be trained by equation S1 with a boundary condition and find the correct solution. Therefore, we set a loss function $l$ to be

| $l=\frac{1}{N}\sum_{i=1}^{N} \left[ \left( \frac{dt^{NN}}{dx} \right)_{i}-\left( \frac{dt}{dx} \right)_{i} \right]^{2}+\left( t_{0}^{NN}-t_{0} \right)^{2},$ | (S2) |
| --- | --- |

where $x$ and $t^{NN}$ are respectively the input and output of the neural network as shown in Fig. S4a, and $N$ is the number of data points between $x=0$ and $x=1$ , which is a domain that we choose. The first term on the right-hand side of equation S2 is for the network to learn the differential equation, whereas the second term trains the network to give a unique solution according to a boundary condition; i.e., $t_{0}^{NN}=t^{NN}(x=0)$ and $t_{0}=t(x=0)$.

The network (i.e., a fully connected network having four hidden layers with 100 neurons and a bias for each layer) is trained to minimize the expression in equation S2. Results (Fig. S4b) for the three boundary conditions, $t_{0}=0, 10$ and 100, show the excellent performance of the network. Here, the black lines are the target functions $t(x)$, the 100 red lines indicate results of the network from the Monte Carlo dropout, which can be regarded as the model uncertainty [3] of the network, and the blue lines are averages of the red lines.

For those readers who are interested in what would happen to the network if a boundary condition is not available, we have prepared the same network (Fig. S4c) using a modified loss function, which excludes the last term in equation S2. Results (Fig. S4d and S4e) are drawn with the same color code as in Fig. S4b, except for the orange lines, whose offset values are artificially removed to make an easy comparison with $t\left( x \right)=\sin\left( 14\pi x \right)$ in black. As anticipated, the network predicts a solution with a random finite offset. We also compared (Fig. S4e) the first and second derivatives of $t^{NN}$ (red and blue lines) with the target functions (black), showing the excellent performance of the network. As a final remark, we expect an offset to be random because there are no explicit constraints on the boundary condition of the network. Nevertheless, we find that a histogram of the random offsets seemingly follows a normal distribution (Fig. S4f) generated for 300 networks. Discussions on why the histogram follows a normal distribution and what determines the mean and variance of the random offsets are beyond the scope of this study.

**
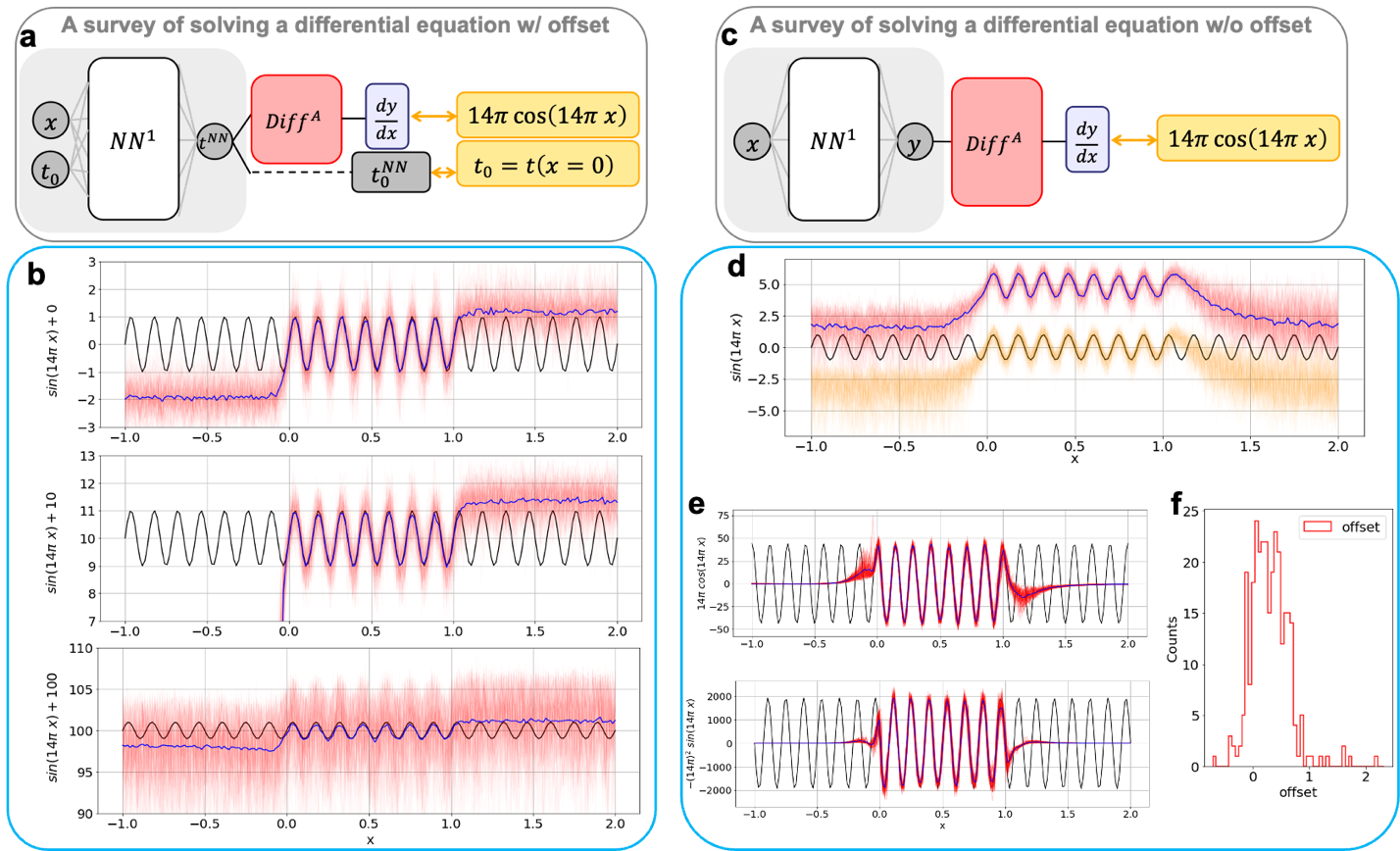
Fig. S4.**

**Neural network solving a differential equation.** (**a**) Schematic diagram of the neural network for learning a differential equation with a boundary condition. ${NN}^{1}$ is the network with four fully connected hidden layers, and ${Diff}^{A}$ performs automatic differentiation to generate the first derivative of $t^{NN}$ with respect to the input variable $x.$ The network is then trained with target answers indicated by the yellow boxes. (**b**) Results (red and blue lines) of the network for the three boundary conditions: $t_{0}=0, 10$ and 100. The blue lines are averages of the 100 red lines obtained using the Monte Carlo dropout. Black lines indicate target functions. (**c**) Same network as in (a) except for the boundary condition; i.e., no boundary condition is imposed on this network, such that there is a random offset $t_{0}^{NN}$. (**d**) Results of the network (red, blue and orange lines) with the same color code as in (b), although the orange lines are artificially offset-removed red lines for easy comparison with a target function (black). (**e**) The first and second derivatives of $t^{NN}$ (red and blue lines) agree well with the target functions. (**F**) A histogram of the random offsets generated by 300 networks in (c) seems to follow a normal distribution.

## **Note S2: Typical KSTAR experimental discharge and its equilibrium characteristics**

We present an example of a typical KSTAR discharge (KSTAR shot #22989) with a succinct description of the operation sequence. A KSTAR experiment first starts with the magnetization stage (time between $t=-15$ and $t=0$ s), where external coils such as the poloidal field coils are charged to their initial target values (Fig. S5b). As the magnetization is fully ready, the plasma is initiated at $t=0$ s and driven to generate the plasma current (Fig. S5a), which generates, in turn, the poloidal magnetic fields inside the tokamak for the confinement of fusion-grade plasmas. The plasma current goes through three distinct phases known as the ramp-up, flat-top and ramp-down phases, where the current is respectively increasing in time, in a steady state and decreasing in time.

During operation, poloidal magnetic fields (Fig. S5c−e) are measured in real time using tens of magnetic pick-up probes and flux loops. The plasma current (Fig. S5a) is measured using a Rogowski coil. Furthermore, the plasma boundary is continuously changing over time as shown in Fig. S5f; therefore, the boundary position constantly during operation needs to be determined.

Figure S5g shows a poloidal cross-section of the magnetohydrodynamic equilibrium at a certain time of the KSTAR discharge with constant contours of the flux surfaces and five important geometrical positions indicated by the red dots. $(R_{0}, Z_{0})$ is the position where the magnetic axis is located. Note that $R_{0}$ is the major radius of the plasma. $R_{min}$, $R_{max}$, $Z_{min}$ and $Z_{max}$ are self-explanatory and determined by the plasma boundary, which is also known as the last closed flux surface where the magnetic X-point resides; i.e., $(R_{Zmin}, Z_{min})$ in Fig. S5g. $R_{Zmin}$ and $R_{Zmax}$ are respectively the $R$-positions of the flux surface at $Z_{min}$ and $Z_{max}$, whereas $Z_{Rmin}$ and $Z_{Rmax}$ are respectively the $Z$-positions of the flux surface at $R_{min}$ and $R_{max}$,. The four most outer positions of the plasma are shown in Fig. S5g as the red dots along the last closed flux surface. We define three other geometrical parameters using these five positions: the minor radius $a=\frac{R_{max}-R_{min}}{2}$ (the half distance between innermost and outermost $R$ positions of a plasma), the elongation $\kappa=\frac{Z_{max}-Z_{min}}{2a}$ (the ratio of vertical to horizontal dimensions of the plasma) and the triangularity $\delta=\frac{(R_{max}+R_{min})/2 -R_{Zmax}}{a}$ (a shaping parameter).


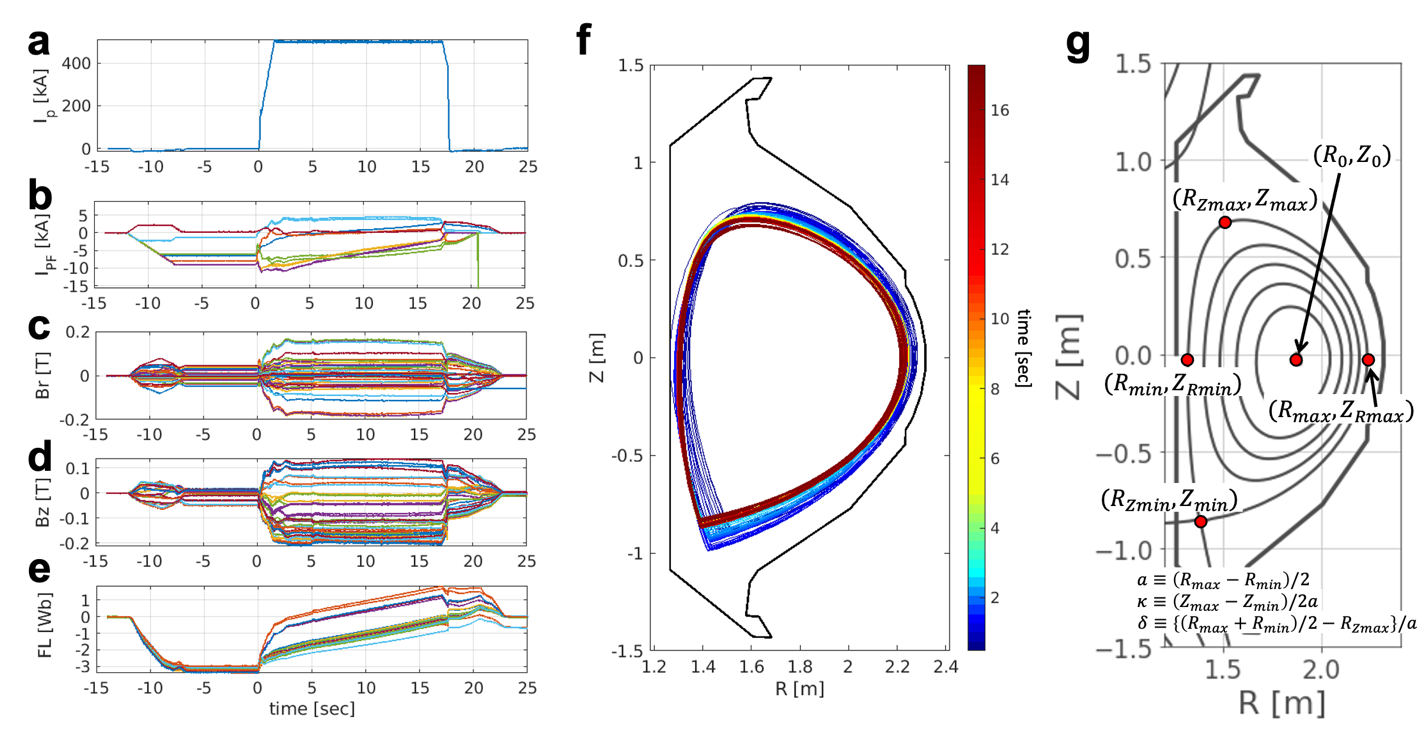


**Fig. S5.**

**Example of the KSTAR discharge (KSTAR shot #22989).** (**a**) Temporal evolution of the plasma current. At $t=0$, the plasma is initiated and driven to generate the plasma current. (**b**) Temporal controls of the currents flowing through poloidal field coils that are commanded according to an experimental plan. Different colors indicate different poloidal field coils. During the initial magnetization stage from $t=-15$ s to $t=0$ s, the poloidal field coils are charged to the initial target values. (**c**−**e**) Magnetic fields $B_{R}$, $B_{Z}$ and $\psi_{FL}$ measured by the magnetic pick-up probes ($B_{R}$ and $B_{Z}$) and flux loops ($\psi_{FL}$) used in this study. (**f**) Samples of the plasma boundary on a poloidal cross-section of KSTAR where different colors indicate different times of the discharge. (**g**) Example of the magnetohydrodynamic equilibrium at a certain time during the discharge with basic control parameters associated with the plasma geometry.

## **Note S3: Conceptual design of GS-DeepNet with kinetic constraints**

We introduce a conceptual structure of GS-DeepNet that constructs the plasma equilibria constrained with radial profiles of the total plasma pressure (including fast ions) and the magnetic pitch angles, often referred to as the kinetic profiles, if they become available in real time in the near future. As discussed in Materials and Methods in the main text, the Force-Balance Net solves the ill-posed problem associated with a large number of unknown current sources $\vec{J}_{\phi}$ by recasting the problem into the matrix form and using the SVD scheme. If the kinetic profiles are available in real time, then we can directly obtain the plasma current density at the $i^{\text{th}}$ grid position as in $J_{\phi,i}=R_{i}p_{i}^{'}+ff_{i}^{'}/R_{i}\mu_{0}$ because $p_{i}^{'}$ and ${ff}_{i}^{'}$ can be estimated from the total plasma pressure $p_{tot}$ and magnetic pitch angle $\gamma_{m}$.

We can add an auto-encoder to accept the real-time available measured $p_{tot}$ and $\gamma_{m}$ and output $p_{tot}^{AE}$ and $\gamma_{m}^{AE}$, which become additional input parameters to the Maxwell Net (Fig. S6). The autoencoder acts to reduce the noise in the measurements as well as to represent the kinetic profiles with possibly some other features so that the Maxwell Net and Force-Balance Net sensitively acknowledge the new information in solving the Grad–Shafranov equation. We leave the implementation of this conceptual network as future work because the measured $p_{tot}$ and $\gamma_{m}$ are not available in real time at the moment.


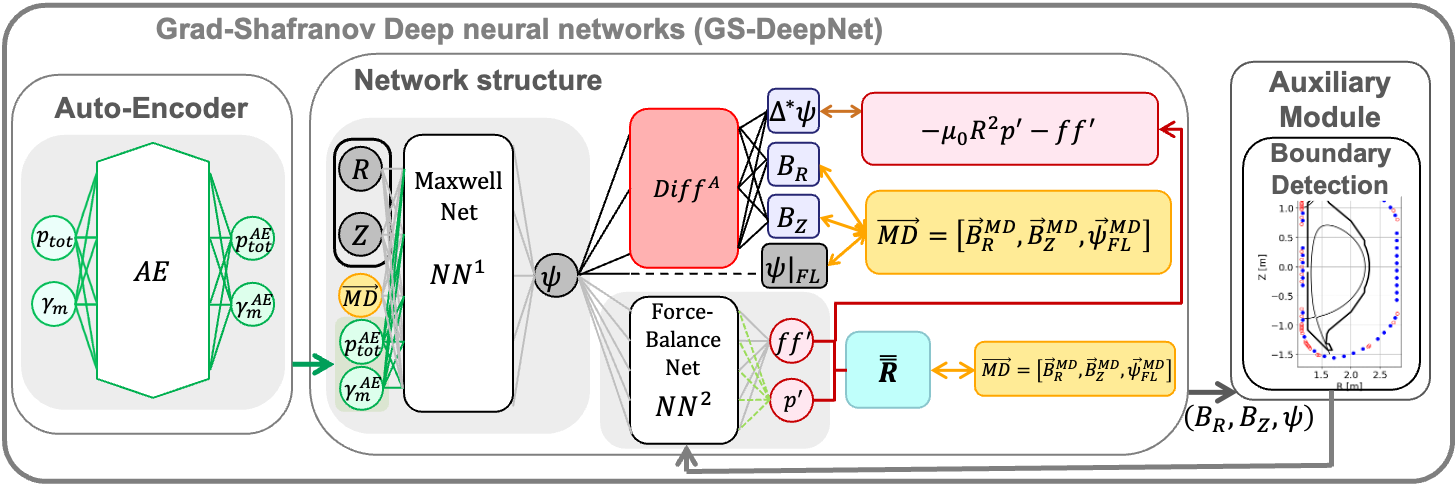


**Fig. S6.**

**Conceptual design of GS-DeepNet with the kinetic constraints.** An auto-encoder is introduced to accept profiles of the real-time available total plasma pressure $p_{tot}$ and the magnetic pitch angle $\gamma_{m}$. The output of the auto-encoder, $p_{tot}^{AE}$ and $\gamma_{m}^{AE}$, contains sensitive features for solving the Grad–Shafranov equation and enters into the Maxwell Net as an additional input parameter.

# Supplementary References

1. Lee, S., Bak, J., Ka, E., Kim, J. & Hahn, S. Magnetic diagnostics for the first plasma operation in Korea superconducting tokamak advanced research. *Review of Scientific Instruments* **79**, 10F117 (2008).
2. Urankar, L. Vector potential and magnetic field of current-carrying finite arc segment in analytical form, part III: exact computation for rectangular cross section. *IEEE Transactions on Magnetics* **18**, 1860-1867 (1982).
3. Gal, Y. & Ghahramani, Z. Dropout as a Bayesian approximation: representing model uncertainty in deep learning. *International Conference on Machine Learning* (2016), pp. 1050-1059.
